# The facilitators of and barriers to antimicrobial use and misuse in Lalitpur, Nepal: a qualitative study

**DOI:** 10.1186/s12889-024-18690-9

**Published:** 2024-05-02

**Authors:** Summita Udas, Obindra Bahadur Chand, Babin Shrestha, Sushmita Pathak, Sarita Syantang, Ashata Dahal, Abhilasha Karkey, Abhishek Giri, Olita Shilpakar, Buddha Basnyat, Olawale Salami, Juvenal Nkeramahame, Piero Olliaro, Philip Horgan

**Affiliations:** 1 Oxford University Clinical Research Unit Nepal, Patan, Nepal; 2 School of Health and Social Care, University of Essex, Colchester, UK; 3 FIND, Geneva, Switzerland; 4 International Severe Acute Respiratory and Emerging Infection Consortium, Pandemic Sciences Institute, University of Oxford, Oxford, UK; 5 Evidence & Impact Oxford, Oxford, UK

**Keywords:** AMR, Evidence-based intervention, Social and behavioural science, Knowledge and awareness, Doctor–patient interaction

## Abstract

**Background:**

Antimicrobial resistance (AMR) is a pressing global health concern driven by inappropriate antibiotic use, which is in turn influenced by various social, systemic, and individual factors. This study, nested within FIND’s AMR Diagnostic Use Accelerator clinical trial in Nepal, aimed to (i) explore the perspectives of patients, caregivers, and healthcare workers (HCWs) on antibiotic prescription adherence and (ii) assess the impact of a training and communication (T&C) intervention on adherence to antibiotic prescriptions.

**Methods:**

Using qualitative, semi-structured interviews, pre-intervention and Day 7 follow-up components, and the Behaviour Change Wheel process, we investigated the facilitators of and barriers to the use and misuse of antibiotic prescriptions.

**Results:**

Results of the study revealed that adherence to antibiotic prescriptions is influenced by a complex interplay of factors, including knowledge and understanding, forgetfulness, effective communication, expectations, beliefs and habits, attitudes and behaviours, convenience of purchasing, trust in medical effectiveness, and issues of child preferences. The T&C package was also shown to play a role in addressing specific barriers to treatment adherence.

**Conclusions:**

Overall, the results of this study provide a nuanced understanding of the challenges associated with antibiotic use and suggest that tailored interventions, informed by behaviour frameworks, can enhance prescription adherence, may be applicable in diverse settings and can contribute to the global effort to mitigate the rising threat of AMR.

**Supplementary Information:**

The online version contains supplementary material available at 10.1186/s12889-024-18690-9.

## Background

In recent decades, antimicrobial resistance (AMR) has proven to be a multi-layered and multifactorial challenge to health systems and their users. According to O’Neill et al. [1], 700,000 deaths per year can be attributed to drug-resistant pathogens, and if no action is taken it is estimated that this number will rise to 10 million per year by 2050. In addition to increasing mortality rates, AMR can cause serious illness, prolong hospital stays, lead to treatment failure, and increase economic burden [2]. Consequently, it poses a serious challenge to achieving universal health coverage [3, 4], achieving Sustainable Development Goals, and upholding the right to health guaranteed by the Constitution of Nepal [5].

AMR has also been identified as a growing public health challenge in Nepal’s Health Sector Strategy 2015–2020 [6, 7]. AMR surveillance was initiated by the Government of Nepal in 1999 to address emerging issues, and the programme has now expanded to 26 participating laboratories located across all seven provinces, including the Central Veterinary Laboratory (CVL) [8]. Although this is a welcome expansion of the AMR surveillance programme in the country, it has not been adequate in addressing the emergence of AMR at the multiple levels required, from local to national. Additionally, the programme has often faced inconsistent, incomplete, and delayed reporting of data to reference laboratories such as the National Public Health Laboratory (NPHL) and CVL [8].

Globally, AMR is primarily caused by the inappropriate use and overuse of antibiotics and failure to follow recommended treatment protocols [9–12, and 13]. In particular, physicians and healthcare providers may inappropriately prescribe antibiotics due to difficulties in diagnosing and treating acute febrile illnesses, especially in low- and middle-income countries (LMICs) such as Nepal [14, 15]. Also, because of challenges in the health system Nepal has struggled to cope with the rising burden of AMR, including a lack of preparedness measures for clean water, sanitation, and infrastructure [15, 16]. This lack of resources hampers Nepal’s health system and service delivery, which in turn has had an adverse impact on doctor-patient interactions and patient treatment/recovery, contributing to poor adherence to treatment [17].

To combat this issue, FIND’s AMR Diagnostic Use Accelerator programme [18] was launched at the AMR Call to Action event in Ghana on the 18th of November 2018. In the first phase of this programme, a five-country clinical trial (Burkina Faso, Ghana, India, Nepal, Uganda) was conducted by the Advancing Access to Diagnostic Innovation Essential for Universal Health Coverage and AMR Prevention (ADIP) group, which also included nested qualitative components.

### Aim of the clinical trial

The primary aim of the five-country clinical trial was to address the following question: in children and adolescents (plus some adults at certain sites) who present to outpatient clinics or peripheral health centres in LMICs with acute febrile illness, will a package of diagnostic tests, diagnostic algorithms, clinical process flows, and training and communication (T&C) for healthcare workers (HCWs) and patients/caregivers improve the case management of acute febrile illnesses, and enable better targeting (i.e., the correct use) of antibiotics compared with current clinical practice?

### Primary clinical trial objectives

The primary objectives of the five-country clinical trial were to assess the impact of the intervention package on (1) clinical outcomes and (2) antibiotic prescriptions compared with routine practice for children and adolescents (plus adults in certain sites) presenting at outpatient clinics.

### Secondary clinical trial objectives

Secondary objectives of the five-country clinical trial were to: (1) assess adherence to the new diagnostic algorithm by HCWs; (2) assess adherence to prescriptions by patients/caregivers; and (3) to evaluate safety outcomes.

### Novelty and unique contribution to knowledge

As noted in a previous publication by Compaore et al. [19], existing systematic reviews have highlighted the limited number of studies examining the behaviour drivers of medicine adherence in LMICs, particularly in Asia [20, 21].

*‘Few studies on compliance have been performed in Asian and developing countries where most of the world’s population resides,’ Jin et al., 2008* [20].

Reflecting this, Schmiege et al. highlighted that systematic review findings are ‘biased towards higher-income countries (HIC) and western countries, highlighting that more evidence is needed from LMICs and other regions.’ [22].

In particular, studies in Asia highlight that the lack of awareness among patients, caregivers, and community members regarding the proper use of antibiotics [23], combined with the health behaviour of individuals and families as shaped by culture, context, and other sociological factors including age, income, occupation, and language, complicate the matter [24]. Together with the limited research available, these factors have posed significant challenges to promoting evidence-based decision making and interventions that would otherwise address complex social and behavioural aspects of health, treatment, and recovery, which could contribute to the control of AMR globally and locally [25, 26]. This paper describes the nested qualitative components of the programme that explored the key facilitators of and barriers to prescription adherence from the perspective of patients, caregivers, and healthcare providers receiving and/or providing treatment for acute febrile illnesses in hospitals in Lalitpur, Nepal. These qualitative components consisted of a pre (clinical) intervention assessment, the results of which informed the design of the T&C intervention, a Day 7 patient follow-up component assessing prescription adherence, and an exploration of interventions that could be used to support prescription adherence. Prescription adherence was defined as obtaining (buying or being given) the prescribed medicine and taking that medicine according to the prescribed instructions for dosage, frequency and duration. For patients who were not prescribed an antibiotic, this meant that they did not subsequently buy or consume an antibiotic.

## Methods

### Study design

In this grounded theory study, we explored behavioural drivers that supported or hindered adherence to prescription using qualitative data collection and analysis. Grounded theory, which is based on inductive reasoning [27], ‘facilitates recording and interpreting individual’s subjective experience’ [28]. Grounded theories ‘drawn from data’ are likely to ‘offer insight, enhance understanding, and provide a meaningful guide to action,’ [29].

The pre-intervention phase of this study was carried out between March and November 2020 and comprised in-depth interviews (IDIs) with patients, caregivers, and HCWs at Patan Hospital, Lalitpur, Nepal (see supplementary documents 1–5). The selection criteria for participants in the pre-intervention qualitative study were similar to those used later in the clinical trial and post-intervention study, and included caregivers of children and adolescents 6 months – 18 years of age and adolescent and adult patients 13 years and older with a fever lasting less than 7 days (a temperature ≥ 37.5°C), either associated with a respiratory tract infection or without any other focus, who were confirmed negative for SARS-COV2, who provided informed consent (or assent for children and adolescents aged > 7 and < 18 years) and committed to a 1-week follow-up. The HCWs selected for the study consisted of members of the Oxford University Clinical Research Unit Nepal (OUCRU NP) outpatient department (OPD).

The findings from these IDIs were then used to develop a T&C package (see supplementary documents 6–15) aimed at enhancing prescription adherence among patients. The package consisted of training for HCWs as well as the clear communication of messages for patients.

To ensure the validity of the training materials, a dedicated scientific committee was established to review the T&C package. The clarity of language in the communication messages was also checked by a small number of community members. The scientific committee was comprised of experts with extensive knowledge of patients and patient behaviours, including the OUCRU NP Director, senior microbiologists, senior consultants, emergency physician and social scientists, doctors and nurses, as well as a clinical trial officer. The scientific committee evaluated the content, methodology and overall quality of the training materials. Their expertise and input contributed to the robustness and reliability of the intervention, enhancing the credibility and validity of the study findings. The final T&C package was then integrated into the clinical trial intervention package.

Before enrolment of any participants in the trial, and as part of protocol training, HCWs in the intervention arm were trained on the delivery of the T&C package. During the conduct of the clinical trial, patients meeting the trial’s selection criteria were randomized (1:1) into an intervention or control arm.

In the intervention arm, patients were examined by a HCW, a series of diagnostic tests were carried out in line with a pre-determined clinical algorithm, and a suitable prescription determined. The communication messages in the T&C package were discussed with the patient or caregiver by the HCW while the prescription was provided. Additionally, a number of communication materials in the consultation space promoted good dialogue between the patient and HCW. In the control arm, patients were assessed as per normal practice and the T&C package was not used.

The Day 7 patient follow-up phase was then carried out between September 2021 and September 2022 at the three sites described in Sect. 2.3. During this phase, additional IDIs were conducted with patients or their caregivers from the clinical trial (see supplementary document 5), to gather data on patient adherence and perceptions of adherence to communication messages. The selection criteria for participants in the pre-intervention and post intervention phases were similar except that patients and caregivers in the post-intervention phase had committed to returning on Day 7 for a follow-up, and had attended one of the study clinics at Patan Hospital, Nepal Korea Friendship Municipality Hospital, or Civil Hospital, Kathmandu.

In this qualitative analysis, we adapted topic guides for language and culture, including IDIs for use in Nepal, from a template used across the AMR programme. The guides were culturally appropriate for use in Nepal and drew on the Capability, Opportunity, and Motivation model of behaviour (COM-B) and the Theoretical Domains Framework (TDF) as described by Michie et al. [30, 31] to support the discussion of behavioural determinants of antibiotic use. The guides were reviewed to ensure clarity and consistency of data collection in Nepal.

### Study sites

The pre-intervention stage of the study was conducted at the ‘Fever clinic’ of Patan Hospital, where patients and caregivers who arrived for treatment for fever symptoms were interviewed.

The Day 7 patient follow-up stage of the study was carried out at three sites: (1) Patan Hospital, Lalitpur; (2) Nepal Korea Friendship Municipality Hospital, Bhaktapur; and (3) Civil Hospital, Kathmandu, Nepal.

### Participants

#### Pre-intervention

IDIs were conducted with 37 patients and caregivers who visited the Fever Clinic at Patan Hospital, and 20 health professionals from the OUCRU NP, between March and June 2020 (Table 1).

**Table 1:** Pre-intervention IDI participants

S.*N*	Participants	Male	Female	Total
1	Patients	10	11	21
2	Caregivers	6	10	16
3	Health Professionals (OUCRU NP)	9	11	20

Abbreviations: Oxford University Clinical Research Unit Nepal (OUCRU NP); IDI, in-depth interview.

From the OUCRU NP, there were sixteen doctors with Bachelor of Medicine or Bachelor of Surgery (MBBS) degrees who had between 6 months to 1 year of working experience. In addition, there were four nurses with Proficiency Certificate Level in Nursing (PCL) and a Bachelor of Science in Nursing (BSN). A total of 17 medical professionals working as medical research officers or nurses from OUCRU NP participated in the survey. Medical research officers were the medical doctors involved in the different clinical research that OUCRU NP was undertaking. Similarly, nurses were those involved in different clinical research that OUCRU NP was undertaking. Overall, the work experience of the medical officers and nurses ranged from 1 to 5 years.

#### Day 7 follow-up (post-intervention)

At Day 7 (± 2), patients were followed-up by telephone or at a health facility to assess clinical outcomes, adherence to prescribed medicines, (e.g., how the prescribed medicine was obtained, medicine dosage, frequency and the duration that instructions were followed) and the intention to adhere to antibiotic prescriptions in the future. All participants (both in the intervention and control arms) enrolled into the clinical trial were specifically asked during the Day 0 visit to report back to the health facility on Day 7 to assess the outcome of their illness. IDIs were conducted with patients or caregivers of patients in the intervention arm and the control arm, as outlined in Tables 2 and 3.

**Table 2:** Day 7 follow-up intervention arm participants

Participant group	Patient/caregiver Male	Patient/caregiver Female
Caregiver (child 0–1 years)	1	3
Caregiver (child 1–5 years)	2	3
Caregiver (child 6–12 years)	0	0
Caregiver (child 12–18 years)	1	1
18–50 years	10	4
> 50	1	0
Total	15	11

**Table 3:** Day 7 follow-up control arm participants

Participant group	Patient/caregiver Male	Patient/caregiver Female
Caregiver (child 0–1 years)	1	1
Caregiver (child 1–5 years)	3	6
Caregiver (child 6–12 years)	0	0
Caregiver (child 12–18 years)	1	0
18–50 years	4	2
> 50	0	1
Total	9	10

### Data collection and sampling

IDIs were conducted by trained social scientists and research assistants. All IDIs in the pre-enrolment phase were conducted face-to-face at Patan hospital. To minimize participant time at the hospital during COVID-19 outbreaks, IDIs during the Day 7 follow-up process were conducted through a mixture of face-to-face and telephone interviews.

#### Sampling of participants and saturation

##### Pre-intervention

The 37 patients and caregivers who visited Patan Hospital Fever Clinic were selected based on attendance at the Hospital Fever Clinic, interest, and availability. Twenty HCWs were also enrolled. All interviews were audio-recorded.

##### Post-intervention (Day 7 follow up)

In the post-intervention phase, IDIs were conducted with all patients or caregivers in the intervention arm and a small number in the control arm, covering adherence to the prescriptions given to them on Day 0 of the study. Information on adherence was then recorded in the patient’s clinical record form for quantitative analysis. As with the pre-intervention phase, interviews were also audio recorded.

From the pool of audio recordings, a sample (see Table 2 for characterization) of 26 patients and caregiver interviews in the intervention arm were selected for analysis by the study social scientist, based on the relevance of the responses to the original research questions, which were then transcribed and analyzed. In addition, 19 patient IDIs in the control arm were selected (based on their willingness to participate and availability), transcribed and analyzed.

##### Sampling saturation

In both pre- and post-interventions, patient and interview recordings were evaluated and coded until data saturation. In the post-intervention process, similar responses were identified after coding 20 transcripts. To ensure that data saturation had been reached, an additional six responses were subsequently coded. No new codes or categories were identified, confirming that saturation had been achieved.

##### Data analysis

During both the pre-intervention and Day 7 follow-up stages, the translators received sufficient consultation and feedback from the research team during the transcribing and translation processes to ensure accurate and high-quality translations.

Thematic analysis was used to analyze the English transcriptions. Initially, the core team members read and re-read the data to develop preliminary codes and emerging themes and sub-theme constructs. These preliminary codes were then discussed and finalized through several rounds of team discussions.

In the pre-intervention phase, the coding was carried out manually using Microsoft Excel and then transferred to NVivo software for further analysis. In the post-intervention phase, coding was carried out manually.

Before coding, the team discussed assumptions and pre-assumptions to ensure a common understanding of the codes used. The coding process was iterative, involving a continuous comparison of codes and constructs with new data, to maintain consistency in coding and develop new codes if required.

The IDI transcripts and coding were then reviewed to identify categories and themes related to the facilitators of and barriers to the access and appropriate use of antimicrobials in the hospital. These were also revised and adapted iteratively.

## Results

### Pre-intervention research themes

Overall, the findings from this study showed that adherence to prescription is influenced by the following:

#### Knowledge and understanding of the use of antibiotics and their benefits

The study interviews highlighted a mixed level of knowledge and understanding, with varying use of antimicrobials by patients and caregivers when they and their children were ill/sick and sought treatment.

Overall, both patients and caregivers broadly understood that antibiotics are medicines used to cure diseases and that they must complete the full course to make a complete recovery. This was reported as one of the motivating factors either for themselves or for their children to adhere to treatment, and to make a follow-up visit to the hospital as suggested by the doctors. Representing the voices of patients who visited Patan hospital, a patient said:

*“If I do not complete the dose there might be relapse… Therefore, to prevent relapse I had to complete dose for curing completely.”* (IDI, male patient).

However, some patients and caregivers said they did not understand much about antibiotics. One patient mentioned:

*“I haven’t understood much about antibiotics. An antibiotic is a very usual medicine. It’s used frequently.”* (IDI, male patient).

Other patients reported that they did not even complete the full course of antibiotics.

*“They instruct us to take it for 3 days. I completed some of it. For example, I didn’t complete it last time because it didn’t cure me, so I thought it was not appropriate to take it.”* (IDI, male patient).

Further, some participants, mostly patients, reported that they do not know much about antibiotics or do not have any knowledge about antibiotics. One of the patients commented:

*“I don’t have actual knowledge about it (antibiotics).”* (IDI, male patient).

In addition, one of the caregivers who had participated in the study mentioned that:

*“Antibiotics should not be bought through self-prescription, and a doctor must be consulted before getting them.”* (IDI, male caregiver).

Lastly, some patients and caregivers perceived that consuming antibiotics could even harm the body.

*“If they prescribe me antibiotics, I immediately get thoughts like: ‘Now, it will cause loss of energy and make it difficult.”* (IDI, female patient).

Overall, while discussing with patients and caregivers their perceptions and their understanding of whether every patient with fever must be given antibiotics, mixed responses were provided. Many of the participants reported that they felt that not every fever patient needs antibiotics, some of them reported that they did not know anything about it, while others mentioned that they were not sure.

#### Patient forgetfulness

Patient forgetfulness was also found to be a major barrier to successful prescription adherence. Patients may forget to take their medication at the right time, or forget to take it altogether, which can have negative consequences for their health.

*“It’s the habit of forgetting that makes things worse when it comes to following compliance.”* (IDI, male patient).

#### Effective communication by the doctor

Doctor-patient interaction was perceived to be a crucial component of the treatment process and the health system.

##### Trust in the doctor’s word

It was found that good communication and trust were essential when it comes to adhering to prescriptions.

*“As per the doctor’s advice, it is told that antibiotics should be taken for 3 days, 5 days, 7 days, 10 days, and 21 days. We shouldn’t leave in the middle. If we leave it in the middle, it could be resistant. The doctor said this. So, we follow accordingly.”* (IDI, male patient).

In addition, doctors reported that they try to make the conversation effective, and make sure that the patients/caregivers understand them.

*“We [tell them] that full course should be taken, otherwise, medicine will not work.”* (IDI, female HCW).

It was also observed that individual characteristics are important contributors to medication adherence.

*“Some of the patients are cooperative. When we tell them once, they note it down. For example, if they follow our instructions, it will be easier for us. However, some of the patients do not try to understand and are stubborn…”* (IDI, female HCW).

One of the patients shared his experience at the hospital:

*“Here doctors don’t explain things clearly… I understand they might be tired of examining so many patients and don’t like to talk too much. In this situation, it’s quite difficult to follow the prescription and medication dosages… when I am confused, I usually ask my friend from the health sector.”* (IDI, female patient).

##### A friendly environment

Our findings also indicated that a friendly environment significantly increases medication adherence:

*“Let’s say, in a hospital if we have a friendly environment and if doctors explain properly about our symptoms/medication then I believe, everyone will follow the prescription.”* (IDI, female patient).

However, not all patients and caregivers have similar experiences in terms of interacting with doctors during their own, or their children’s, treatment. Patients and caregivers reported that changing doctors (visiting different doctors during follow-ups) made patients uneasy as they were interacting with (new) doctors.

*“While coming for follow-up, there will be a different doctor, so we must explain again. It feels quite uneasy.”* (IDI, female patient).

Further, some patients stated that sometimes doctors get angry with the patients or with the caregivers, which has an adverse impact on the doctor-patient interaction. One of the participants commented:

*“I have experienced doctors getting angry and not explaining things clearly when I came for mother’s check-up and my own check-up.”* (IDI, female patient).

On the other hand, doctors mentioned that communication with patients is sometimes challenging if they are sick, as they are often in a state of agitation. One of the doctors stated:

*“It is difficult to communicate because when someone gets sick, he/she will be obviously in a panic state and the only thing they want is to recover fast… sometimes it takes time, and some patients have to be kept in the examination for few days.”* (IDI, female HCW).

##### Language barriers

Language barriers between healthcare providers and patients increasingly affected adherence to prescriptions.

*“Sometimes it’s quite difficult to interact with patients or with the caregivers when they don’t understand the Nepali language clearly….in that case, we take the help of other staff who understand their language.”* (IDI, female HCW).

##### Literacy and education of the patient

It was reported (mainly by doctors) that the educational background of the patients and caregivers contributed to making communication between the doctor and patient more effective.

*“Each and every kind of patient, including educated and uneducated, visits the hospital. Our duty is to explain to them properly about the prescription, side effects, dosage, etc. … It’s easier to explain to educated patients/caregivers in comparison to uneducated…”* (IDI, female HCW).

Another doctor added:

*“I think educational background also makes a slight difference. Even though several people have poor educational backgrounds, they understand when it is explained in layman’s terms. Sometimes, I have seen patients nodding their heads as if they have understood everything, they pretend to be understanding things and when they come for follow-up, we realize that they have not understood anything.”* (IDI, male HCW).

##### Time constraints

Limited time to see patients was reported as one of the key factors that can negatively affect patient-doctor interactions in a clinical setting, and this is also likely to have had an adverse impact on adherence to prescriptions. This applied to patients, caregivers, and doctors.

*“Sometimes, some patients are in so much hurry that they don’t want to listen to us. However, we explain and do whatever we can do from our side.”* (IDI, male HCW).

### Recommendations based on the pre-intervention analysis

Based on the findings of the pre-intervention study, the following recommendations were made to promote treatment adherence and improve doctor-patient-caregiver interactions, in order to strengthen health services and improve healthcare delivery at the hospital.

1. Standard awareness programme(s) regarding the appropriate use of antibiotics should be developed and implemented. Understanding and perceptions differed considerably between individuals and a few even reported that they do not know what antibiotics are and/or why people should complete a full course of treatment. Also, a few patients reported that they believed consuming antibiotics would make people weaker. As such, it is recommended to develop and design an awareness programme to promote a basic knowledge and understanding of antibiotics, taking into consideration the socio-demographic status of patients, e.g., those who might be illiterate, poor, or elderly. This would not only increase awareness among patients and caregivers about the use and misuse of antibiotics, but would also work towards preventing AMR.
2. In addition, easy, innovative, and effective tools/methods should be developed and implemented to remind patients or caregivers to take their treatment, as many patients and caregivers reported that their forgetfulness had an adverse impact on treatment adherence. Doctors should also be trained in effective and efficient modalities of professional communication.
3. Our findings have shown that the interaction between doctor, patient, and caregiver is influenced by several factors including language barriers (two different languages), patient and caregiver illiteracy, fear of patients, attitude, and time (that the doctor is able to spend with every patient). Thus, it is recommended to design and develop innovative and effective communication strategies (such as using simple and everyday language while interacting with patients and caregivers) to help doctors communicate with the patients, which takes into consideration the socio-demographic status of patients and caregivers.

Overall, recommendations 2 and 3 (tools, methods and training) were built into the design and implementation of the T&C package.

### Post-intervention research themes

#### Knowledge about antibiotics

Initial analysis of the interviews showed that both patients and caregivers tended to administer antibiotics either to themselves or their children based on the prescriptions of doctors. Further analysis suggested that several other factors can influence antibiotic use: from the prescription process to individual beliefs and behaviours.

In both the intervention and control arms, patients and caregivers showed different levels of knowledge and understanding of antibiotics, which affected how they handled and administered these drugs.

##### Knowledge of antibiotic use

Patients had varying knowledge and opinions about antibiotics. One of the patients who participated in the study in an intervention arm explained his/her understanding of antibiotics as:

*“My knowledge of antibiotics was based on hearing that they should be used in cases of high fever.”* (Patient, Intervention Arm).

##### Knowledge and beliefs in the importance of adherence and completing the full course

It was found that many patients and caregivers in both the control and intervention arms usually followed doctors’ prescriptions because they believed that being adherent to antibiotic treatment regimens contributed to the full recovery of their children or themselves. A caregiver at the control arm noted:

*“I give complete medication to my child. I think that the body should get a complete dose of medication because inappropriate dose practice may relapse the disease.”* (Caregiver, Control Arm).

Furthermore, in both intervention and control arms, patients and caregivers noted that doctors explained the duration and course of medications extensively. One of the caregivers from the intervention arm explained:

*“I liked the information provided from here (OPD). Even while going [home], I was very glad [thinking] there should be a facility at the hospital. They wrote it all properly and said to take this [medicine] at a given time. Before there was no such communication, instead they (the doctor) just asked us to get the medicine as prescribed. But now I am happy they had written everything properly.” (Patient, Intervention Arm)*.

Patients and caregivers reported that these communications enabled them to complete the full dose.

*“Doctors had prescribed medicine for 3 days. My child recovered in 2 days. However, what [the doctor] had told me was, “You must complete the course even if she recovers.” So, I gave it in the morning of the third day as well.”* (Caregiver, Intervention Arm).

##### Importance of follow-up

Similarly, interactions with doctors enabled patients and caregivers to handle antibiotics properly and visit the hospital for follow-up in a prescribed manner. As such, they (patients and caregivers) were able to adhere to the prescriptions more easily. For instance, a patient in the control arm explained:

*“The doctor prescribed me to gargle and take paracetamol before bed. If I had a problem, he told me to come back on Thursday. This is why I came today.”* (Patient, Control Arm).

#### Expectation

Patients’ expectations can play an important role in medication adherence, and according to both patients and HCWs, healthcare providers should take steps to manage those expectations and support patients in adhering to their medication regimen.

Our analysis highlighted that patients and caregivers have many expectations, particularly from doctors, hospitals, and larger health systems. Most of the patients and caregivers in the control and intervention arms expected to receive efficient and effective treatment from the doctors, and expected that the doctors would have time to listen to their queries and questions. They also preferred good communication and behaviour from the doctors, as some had previously experienced doctors being rude and not explaining their prescriptions and problems properly.

##### Efficient treatment

One caregiver stated that healthcare providers can help patients feel empowered and engaged in their own care, which can ultimately lead to better adherence to their prescription and improved health outcomes:

*“Especially when my child is in a difficult condition, I am pleased when the doctor provides treatment efficiently. Effective and compassionate care not only improves my child’s health but also fosters trust in the medical team. When patients receive good treatment, they are more likely to adhere to their prescribed therapies and follow medical advice, leading to better outcomes and overall well-being.”* (Caregiver, Intervention Arm).

##### Thorough examination without negligence/time factors

Patients felt that doctors should take the time to explain the purpose of each medication or treatment, the expected outcomes, and any potential side effects or risks. They also felt they should encourage patients to ask questions and express any concerns they may have. Moreover, while explaining what they expected from doctors or hospital care, a patient in the control arm mentioned:

*“The doctor should thoroughly examine us and listen to our questions. If I keep asking more questions, the doctor might become angry since he is very busy. Besides their busy schedule my expectation is that he should clear my doubts…”* (Patient, Control Arm).

A caregiver in the intervention arm also noted:

*“Doctors should prescribe medicine through proper check-ups and examinations. They should explain properly about the prescription.”* (Caregiver, Intervention Arm).

##### Nice behaviour

Patients believed that if doctors displayed a kind and empathetic attitude toward them, it could help improve medication adherence. Additionally, polite and friendly behaviour by doctors was one of the expectations of patients and caregivers. A patient from the control arm stated:

*“I want the doctor to speak politely to me. The patient feels less pain if doctors behave well. Doctors sometimes need to show sympathy to their patients.”* (Patient, Control Arm).

##### Explain clearly or good communication

In addition, doctors were expected to use clear and simple language when interacting with patients and caregivers. A caregiver from the control arm noted:

*“I believe doctors should explain things clearly so patients can understand them. Patients often complain that they don’t understand what their doctors say. The doctors should take such things into consideration since it will simplify interactions and make communication understandable for all—the doctors and patients.”* (Caregiver, Control Arm).

##### Examination of old medical records

Finally, the examination of old medical records was another expectation of patients and caregivers. One of the patients from the control arm expressed his/her expectations of doctors as:

*“I usually wish the doctor checks and analyses all the old records properly, [and] explains the new problems linking with the old records.” (Patient, Control Arm)*.

#### Beliefs and habits

Our analysis also highlighted that patients’ and caregivers’ beliefs were an important factor influencing adherence to medication. For example, some patients believed that antibiotics make people weak and that medicine can also be used as a fertilizer.

##### Taking a full course of antibiotics leads to recovery

In both intervention and control arms, patients generally understood that completing the course of antibiotics led to complete recovery:

*“I think the regular use of medicine will reduce illness, but taking the complete course will make you fully recovered.”* (Patient, Intervention Arm).

*“I give complete medication to my child. I think that the body should get a complete dose of medication because inappropriate dose practice may relapse the disease.”* (Caregivers, Intervention Arm).

##### Antibiotics make people weak

However, some patients and caregivers believed that antibiotics could cause weakness. One of the patients in the intervention arm mentioned:

*“I have heard that taking antibiotics could make people weak or with low energy.”* (Patient, Intervention Arm).

##### A habit to throw leftover medicine and use it as fertilizer

Our findings indicated that the knowledge and beliefs of the patients and caregivers may guide their behaviours and practices, particularly relating to the consumption and management of antibiotics. One of the caregivers from the intervention arm explained how they managed their remaining antibiotics by stating:

*“We throw leftover medicine [away] or we generally pour them in a flower vessel or in a garden, thinking that flower will bloom nicely.”* (Caregiver, Intervention Arm).

Similar responses were seen in the control arm. For example, during a conversation with a caregiver about remaining medicine, they said:

*“We throw remaining medicines as its date might expire and it might get misplaced as well.”* (Caregiver, Control Arm).

#### Behaviours

##### The behaviour of the doctors

The behaviour of doctors was also reported to be a key influential factor for both treatment adherence and hospital follow-up attendance. Doctors’ friendly behaviour led to comfortable communication and a better understanding of prescriptions. Conversely, rude behaviour from doctors could de-motivate patients or caregivers and influence whether they continued medication and treatment. A patient in the intervention arm explained:

*“I don’t go for follow-up due to the doctor’s rude behaviour.”* (Patient, Intervention Arm).

##### Self-determination

Most patients and caregivers, in both control and intervention arms, decided to stop taking or keep taking medications on their own without consulting their doctors. “I decided myself,” was a phrase we heard repeatedly from patients or caregivers when we asked why they chose to stop medications or why they continued to take them. For many medications, after a slight recovery, they tended to stop taking them as prescribed. A patient from the control arm mentioned:

*“In the case of antibiotics, we have been giving it until the recommended days but in the case of non-antibiotics, we usually don’t complete the dose and after slight recovery stop consuming.”* (Patient, Control Arm).

#### Buying the prescribed medicines

Several factors were also identified that could affect where and why medicine was obtained. These included convenience, trust in medicine quality, and costs.

##### The convenient place for buying medicines

During our discussion with patients and caregivers about convenient places to buy medicines, most reported that they had bought their medicines at the hospital pharmacy, and only a small percentage mentioned that they had purchased their medicines elsewhere. A caregiver in the control arm mentioned that the reason for buying medicine at the hospital pharmacy was convenience:

*“Buying medicine from the hospital is convenient as we had the check-up here as well.”* (Caregiver, Control Arm).

However, in the intervention arm, some of the patients mentioned they had bought medicines from vendors near their homes, as this was more convenient for them.

*“I don’t remember the exact location of the medical shop, but it is near Thimi. The medical [shop] is situated on the way to my home and it’s nearby so I bought from there.”* (Patient, Intervention Arm).

##### Trust in medicine effectiveness

Other patients/caregivers preferred to buy medicine from the hospital as they believe that the medicine produced there was safe and of good quality.

*“I bought medicine for my child from outside on my last visit, but it was not effective. I realize that the medicine that I took from ... the hospital pharmacy is quite effective. So, I come here only to get the medicine though it is far from my house.”* (Caregiver, Intervention Arm).

##### Costs

Patients and caregivers also found that medicines at hospital pharmacies were comparatively cheaper than other pharmacies. A patient mentioned that the main reason to buy medicines at a hospital pharmacy was due to cost:

*“Usually, I buy medicine from the hospital pharmacy. It’s less expensive than outside.”* (Patient, Intervention Arm).

#### Facilitators of and barriers to completing a full course of medicine

Our findings have highlighted several barriers to completing the full course of prescribed medicine, including patient forgetfulness, age, lack of knowledge in distinguishing between antibiotics and non-antibiotics, and a tendency to discontinue medicine after recovery.

Patients and caregivers in both arms often used antibiotics in an arbitrary manner due to different levels of knowledge and understanding. Indeed, many patients and caregivers stopped taking antibiotics in the middle of the course.

##### Forgetfulness

A key reason that was identified for not completing the medication course was forgetfulness:

*“I forget to take medicine. I only remember it when I am in pain.”* (Patient, Control Arm).

##### Communication problems

Many of the reasons for not adhering to medication regimens were similar in both the control and intervention arms. For example, communication problems between doctors, patients, and caregivers, as well as forgetfulness, were commonly reported. One of the patients from the control setting noted:

“I could not convey to the doctor properly as I could not speak in Nepali language. I speak the Newari language due to which I found it difficult to follow the prescription.” (Patient, Control Arm).

##### Discontinuation after recovery from symptoms

Discontinuation of medication after recovery from symptoms was a major barrier to adherence that was commonly reported among patients. For example, one patient described her experience as:

*“Sometimes, I discontinued the medicine after a slight recovery.”* (Patient, Intervention Arm).

##### Children - challenges

In some cases the age of the patient, e.g., a child, was a key factor affecting adherence to treatment and could even led to discontinuation of treatment. A caregiver from the intervention arm stated:

*“When I try to force my child to take the medicine, he becomes angry. Despite my best efforts, I gave up (discontinued) due to his behaviour.”* (Caregiver, Intervention Arm).

As it can often be challenging to make children swallow antibiotics, parents/caregivers seek innovative ways to facilitate the process. Caregivers, for example, put antibiotics in children’s food. A caregiver in an intervention setting explained how she administered medicine to her child:

*“My child hated taking medicine, so I mixed it with nutritious porridge, and he was able to take it.”* (Caregiver, Intervention Arm).

### Reported impacts of the T&C package intervention

The T&C package aimed to facilitate communication between healthcare providers and patients in clinical settings, educate them on the appropriate use of antibiotics, and indicate where they can be bought. The analysis of the interviews revealed that patients and caregivers had a mixed (both positive and negative) experience of the T&C package. However, both patients and caregivers felt that the T&C package contributed to promoting adherence to treatment among patients. Participants also mentioned that the medicine bag was not only practically convenient, but that it served as a reminder to take or administer the medication, thereby improving medication adherence amongst patients through knowledge and awareness.

#### Medication reminders note

Patients found the medication reminder note an effective tool for improving adherence to treatment. Patients often thought they would forget to take their medications, and medication reminder notes helped them to take their medications on time and to take the correct dose.

“*I used to feel like I would forget [taking medicine]. However, it didn’t happen. They had sent written [note] about taking medicine on time.”* (Patient, Intervention Arm).

#### Message in the medical bag

Both patients and caretakers participating in the study found that a message written on a bag (e.g., in the intervention package) served as a visual cue to remind them of the importance of sticking to their medication regimen and felt it helped improve adherence. A patient from the intervention setting explained:

*“I found a message beneficial, and I felt that I should have learned it before. It’s a very usual and important thing; one should share with others after being self-aware.”* (Patient, Intervention Arm).

#### Information and educational material about antibiotics (AMR booklet)

Both patients and caregivers also found the AMR booklet useful and a beneficial tool. They mentioned that, with the help of the booklet, they knew more about antibiotics, their uses, and their importance.

*“The booklet about antibiotics was a good source of knowledge and information.”* (Patient, Intervention Arm).

*“Everything is good; information was about medicine dose that was helpful.”* (Patient, Intervention Arm).

Overall, it was found that information provided on Day 0 was beneficial and improved communication around antibiotics and their use, as well as increasing knowledge and awareness of the importance of antibiotics and adhering to the prescribed treatment regimen. For example, one of the patients mentioned that:

*“I now understood that antibiotics should be taken for the full course.”* (Patient, Intervention Arm).

#### Storage of medicine

The T&C package medical bag also served as a place to store medicine for some patients and caregivers, enabling them to locate their medicines efficiently and administer them on time. The patients or caregivers made sure to keep the medical bag in visible places, where it helped to remind them to take the medicine. Acknowledging the multiple uses of the medicine bag, one of the patients stated:

*“After getting a medical bag (referring to the T&C package), I stored my medicine there, it was very useful and effective as it reminds me to take medicines regularly and properly.” (*Patient, Intervention Arm).

#### Familiarity with doctors

Doctor-patient interactions were effective when the patients were familiar with the doctors, as a patient noted:

*“It was good. I had worked here before, and I know it well. I used to talk to every doctor; they were like friends to me. They used to behave like friends from the beginning. It was easier for me to talk with them.”* (Patient, Intervention Arm).

Overall, as well as the insights provided by the interviews, improvements were identified that could make more effective use and scalability of the T&C in the future. For example, the information included in the T&C package should consider the diverse level of understanding of the patients and caregivers, so that any information provided is useful for both those who have some knowledge and awareness of the prevention and control of AMR, as well as those who may have limited knowledge and understanding. However, it should be remembered that this additional information may not be useful for all patients and caregivers, as busy caretakers who care for more than one patient at home may not get time to read messages written on the medical bag, while others may not find the information new and/or informative.

## Discussion

### Reflection on results

Broadly speaking, the factors that hindered or supported adherence to prescription identified in the pre-intervention study were also present in the post-intervention study, albeit with additional and more in-depth details. Additionally, the findings indicate that a collaborative effort between health and social sciences can provide valuable insights into the wider determinants and drivers of health, specifically relating to AMR challenges in low resource settings like Nepal. Thus, these collaborations and research can contribute to the development, design and implementation of culturally-appropriate and contextually-relevant interventions to address the existing gaps in the health system.

The pre-intervention study identified the following major themes that affected prescription adherence: the knowledge and understanding of individual patients and caregivers; the level of effective communication; the level of trust in doctors; the friendliness of the clinical environment; the ability to communicate in a common language; the literacy of patients; and having sufficient time for consultations.

The post-intervention study identified the following factors that affected prescription adherence: knowledge about antibiotics (e.g., antibiotic use, importance of completing the full course, follow-up); expectations (efficacy of treatment, thorough medical examination without negligence/time constraints, clear communication, appropriate utilization of patient medical records), beliefs and habits (antibiotics make people weak, throwing away leftover antibiotics), behaviour (of doctors and patient/caregivers [self-determination]), buying the prescribed medicine (convenience of the vendor’s location, trust in medicine effectiveness, costs), facilitators of and barriers to completing the full course of medicine (forgetfulness, communication problems, discontinuation after recovery from symptoms, challenges with administration to children).

These findings help to fill the knowledge gap around behaviour drivers for prescription adherence, in LMICs and more specifically in Nepal.

In both pre- and post-intervention we identified similar themes to those reported in previous systematic reviews, as well as those reported for high and upper-middle income countries, such as the importance of cost and income, patient knowledge, and the fulfilment of prescriptions [22]. However, these systematic reviews did not emphasize the specific beliefs identified in our study (e.g., that antibiotics could make people weak), or capture the context-specific importance of each driver. For example, it was notable that the relationship and two-way communication between HCWs and patients was a significant factor in prescription adherence, and was perhaps more significant than issues of cost, which had been emphasized as a key behavioural driver in other LMIC studies [19].

### Implications

Overall, the findings from this study highlight the suitability and benefits of an HCW-patient T&C intervention to support adherence to treatment and suggest that interventions could be developed to address specific barriers to treatment in other areas.

Additionally, the research suggests that a collaborative effort between health and social sciences could shed light on wider determinants and drivers of health, specifically dealing with AMR challenges in low resource settings like Nepal. As such, it is hoped that these collaborations and research could contribute to the development, design and implementation of culturally-appropriate and contextually-relevant interventions to address the existing gaps in the health system. However, considering the scope of the study it is not known to what degree the behavioural determinants, and hence the messages in the T&C package, are likely to differ in other socio-economic settings in Nepal outside of the Kathmandu valley.

#### Recommendations from the behaviour change wheel process

This study used the Behaviour Change Wheel process (BCW) [30, 31] to investigate the design of future interventions to support adherence to prescriptions, based on themes identified in the pre- and post-intervention analyses. The BCW process uses behavioural frameworks to help design interventions based on the categorization of required behaviours and their drivers. We used the BCW process to categorize the themes identified in Day 7 research into the COM-B and TDF frameworks, using the embedded APEASE criteria (Acceptability, Practicability, Effectiveness, Affordability, Side-effects, and Equity) to identify appropriate behaviour change techniques and future intervention designs.

Overall, the barriers for adherence to prescriptions fell into one of the following three topics:

##### Paediatric population non-adherence to prescription

We found that paediatric medication non-adherence was a major problem. Medications administered to children exist in a variety of different formulations, including solids, powders, suspensions, liquids, and topical forms. Different formulations also have differing effects on adherence. Children can object to an oral medications’ taste, smell, or even texture. Patients also discontinued medication due to side effects, and factors such as forgetfulness and busy parent schedules led to missed doses.

To address these issues, we recommend that healthcare providers provide clear information to the parents, caregivers, and children about the child’s disease and treatment plan, the prescribed medicine doses, and the medicine’s potential side effects, on an ongoing basis. We also recommend that healthcare providers provide reminder charts/tips to help them take their medicine on time, such as setting the alarm on their phones. It was found that many of the patients and caregivers didn’t know the correct administration techniques for some formulations, so we recommend healthcare providers also provide technical advice on the administration of the medicines.

We also recommend that education is provided through healthcare providers and pharmacists on medicines through individual counselling, complemented with printed leaflets providing information about the prescribed medicines (for the patients), and outdoor posters for the general community. This should take place in hospital settings (OPD, wards, paediatric department) and at community and hospital pharmacies.

This behaviour determinant falls within the COM-B category of ‘psychological capability’ and the TDF categories of ‘knowledge’, ‘memory, attention and decision processes’, and ‘behavioural regulation.’ Similarly, it also falls under ‘physical opportunity’ within the COM-B category and the TDF category of ‘environmental context and resources.’ The recommended intervention uses the behaviour change techniques of information about health consequences, instruction on how to perform a behaviour, and adding objects to the environment.

#### Doctor-patient communication

Effective communication was found to be critical for patient adherence to prescriptions. However, there were many barriers to good communication between doctors and patients, such as non-disclosure of information, lack of attention paid to patient queries/doubts, and discouraging patients from voicing their concerns and expectations.

To address these barriers, we recommend a periodic training intervention that provides hospital management, respective OPDs, healthcare teams, and clinics with effective communication skills. The training should be conducted face-to-face, both for individuals and for groups from the OPD, wards, and hospital/community pharmacies, and should provide a demonstration of appropriate communication and behaviour.

Good communication between doctors and patients could also help to regulate the patient’s emotions, make it easier to obtain the necessary medical information needed for appropriate treatment decisions, and allow for better identification of the patient’s needs, perceptions, and expectations. We therefore recommend that healthcare providers create an environment for the patient to express their feelings without hesitation, and to use prompts and cues within the consulting room to inform and remind patients and doctors of this expectation.

This recommended intervention falls within the COM-B category of ‘psychological capability’ and the TDF category of ‘knowledge, cognitive and interpersonal skills and behavioural regulation.’ The recommended intervention uses the behaviour change technique of demonstrating the behaviour, then restructuring the physical environment.

#### Patient’s behaviour

During this study, it was reported that patients did not take medications when they have limited knowledge about antibiotics, were forgetful, or feared side effects. However, patients also self-medicated (without contacting a medical professional) and took leftover medicine that had been stored.

To address this issue, we recommend an education intervention. Patients/caregivers who visit hospitals and pharmacies should be educated by healthcare providers to raise awareness about antibiotics, their use, the consequences of self-medication and inappropriate dosing practices; this process should be ongoing.

In addition, we recommend training for the doctors. Training should enable doctors to provide counselling to patients about the consequences of using leftover medicine. The training should be given by senior health professionals and hospital management and should be conducted periodically.

We also recommend modelling as an intervention, using drama scenes that reflect the impact of self-medication, enacted by people who are trusted in the community. The intervention can also be planned periodically in the community.

This behaviour determinant falls within the COM-B category of ‘psychological capability, reflective motivation, and physical opportunity,’ and the TDF categories of ‘knowledge’, ‘memory, attention and decision processes’, ‘behavioural regulation’, ‘beliefs about consequences’, and ‘environmental context and resources.’ Similarly, the recommended intervention uses the behaviour change technique that involves demonstration of the behaviour, behavioural practice/rehearsal, and providing information about health consequences.

### Limitations

Most of the fieldwork for this study was conducted during the COVID-19 pandemic, which may have influenced the interviews and overall findings. For example, participants were less expressive compared with non-pandemic settings, due to fear of being infected with COVID-19 while interacting with the interviewer(s). This fear of infection may have been further exacerbated by the interviewers wearing protective gear and requesting that the participants wear a mask while having their conversations. Overall, we felt that the situation did not make them feel comfortable and lead to a natural response, at least at the beginning of the conversations. Due to COVID-19 pandemic restrictions, Focus Group Discussions that were originally planned with health professionals were replaced with virtual IDIs (by telephone), with appropriate and recommended safety measures. However, these virtual interviews did not allow the evaluation of participants’ body language, facial expressions, and overall comfort.

## Conclusions

In conclusion, there are a range of factors that can influence adherence to antibiotic prescriptions, including patient/caregiver knowledge, their expectations of treatment, clear doctor-patient-caregiver communication, patient/caregiver beliefs and habits, the behaviour of doctors and patient/caregivers, and challenges associated with administering medications such as forgetfulness and/or administering medication to children. In addition, the findings from this study demonstrate the suitability and benefits of an HCW-patient T&C intervention to support adherence to antibiotic prescriptions, and suggest that interventions could be developed to address specific barriers to treatment in other areas.

### Electronic supplementary material

Below is the link to the electronic supplementary material.

Supplementary Material 1

Supplementary Material 2

Supplementary Material 3

Supplementary Material 4

Supplementary Material 5

Supplementary Material 6

Supplementary Material 7

Supplementary Material 8

Supplementary Material 9

Supplementary Material 10

Supplementary Material 11

Supplementary Material 12

Supplementary Material 13

Supplementary Material 14

Supplementary Material 15

## Data Availability

The datasets used and/or analyze during the current study are available from the corresponding author on reasonable request.
