# Supplementary material for: The facilitators of and barriers to antimicrobial use and misuse in Lalitpur, Nepal: a qualitative study
Source: BMC Public Health. 2024 May 2;24:1219. doi: 10.1186/s12889-024-18690-9 (PMC11067172; doi:10.1186/s12889-024-18690-9)
Supplement: Supplementary file 1 — Supplementary Material 1 [file 12889_2024_18690_MOESM1_ESM.docx]

**Supplementary File 1. Caregivers/ Patient: Interview Guide**

Interviewer greets the candidate, giving his/her name and position to participants

Thank you for volunteering to take part in Interview. We are very interested to hear your valuable opinion. You have been asked to participate as your point of view is important. I realize you are busy and I appreciate your time.

Introduction: This Interview is designed to assess your current thoughts and feelings about the medicine that you purchase and how they are used. The information you give us is completely confidential, and we will not associate your name with anything you say in the Interview. I would like to audio tape the interview so that we can make sure to capture the thoughts, opinions, and ideas I hear from you. No names will be attached to this session. May I tape the discussion to facilitate its recollection? (If yes, switch on the recorder). I would also like to inform you that I will be taking notes throughout the interview.

Anonymity: Despite being audio taped, I would like to assure you that the discussion will be anonymous. The tapes will be kept safely in a locked facility until they are transcribed word for word, then they will be destroyed. The transcribed notes of interview session will contain no information that would allow individual subjects to be linked to specific statements. You should try to answer as truthfully as possible. If there are any questions or discussions that you do not wish to answer, you do not have to do so; however please try to answer as possible. You may refuse to answer any question or withdraw from the study at anytime.

- Logistics:
- Interview will last about one hour
- Establish Rapport:
- Try not to reveal approval or disapproval, agreement or disagreement through your words, gestures or facial expressions.
- Do not challenge answers or engage in debate with the Interviewee.
- Keep your body language neutral and make notes continuously.
- Nod, smile and encourage the candidate throughout the interview.
- Use silence (up to 10 seconds) to encourage candidates to expand on short answers; ask if they would like to say any more or if they would prefer to move on

Pace the Interview

- If there is a clock in the room, be sure the candidate can see it clearly.
- Gently steer the discussion to the next question if the candidate exceeds allotted time for a particular question.

Probe and Follow‐up

- Prompt the candidate to obtain missing or additional information. Use general questions such as “Can you please tell us a little more about that?” and “What did you do next?”
- If candidates have difficulty thinking of an example, ask them to think about an example they have already provided and consider its relevance.
- Candidates can always return to a question and expand on their answers if time permits.
- Take Notes
- Don’t be selective in your note‐taking—write everything down.
- Take notes of factual data (example, quotes from candidate), not your judgments.
- Conclusion
- Thank you for participating. This has been a very successful discussion
- Your opinions will be a valuable asset to the study
- If there is anything you are unhappy with or wish to complain about, please contact the local PI or speak to me later
- I would like to remind you that any comments featuring in this report will be anonymous

Please, write your report based on the results of Interview. Please remember to maintain confidentiality of the participating individuals by not disclosing their names.

- **Materials and supplies for Interview**
- Consent forms (one copy for participants, one copy for the team)
- 1 recording device

Caregiver questionnaires for in-depth interview

**Demographic data**

1. Interviewers Name
2. Questionnaire number
3. Date
4. Address of interview participants
5. Health Care Facility Name that the participant normally attends
6. What is your relationship to children you take to the health facility?

**Screening question/Doer-non doer**

1. Role: Do you have responsibility to
2. Take children to the clinic when they have fever
3. Buy medicine for children
4. Ensure children take medicine

(Screen out respondents that are not responsible for any of the three actions)

1. Did any of your children have fever in the last 6 months?
2. If yes,
   1. Where did you take them for treatment
   2. Did the children take medicine for the fever?
   3. Prompts
      1. Did you take child to the clinic when they have fever
      2. Did you buy medicine for children? Where?
      3. Did you ensure children take medicine?

**General questions**

1. When you take your child to the Patan Hospital with fever, what makes it easier/would make it easier for you to
2. Buy the medicine that the doctor prescribes
3. Ensure that the child takes the medicine following the instructions from Patan Hospital
4. What makes it difficult to
5. Buy the medicine that the doctor prescribes
6. Ensure that the child takes the medicine following the instructions from Patan Hospital

Normal behavior

The last time that your child had fever and you took them to Patan Hospital

- Were you prescribed medicine? YES/NO
- Did you buy medicines as instructed by physician? YES/NO
  - Why__________________________________________________________
- What medicines were you given/did you buy?

___________________________________________________________

- Did you complete antibiotics as prescribed by the doctors YES/NO/NA
  - Why________________________________________
- Did you buy antibiotics if not prescribed by the doctors? YES/NO/NA
  - Why_________________________________________
- Did you come for a follow-up as instructed? YES/NO/NA
  - Why__________________________________________________________

*NA- not applicable

Capability

Knowledge

- Do you know what antibiotics are, and what they are used to treat? YES/NO

(If not to be explained by the interviewer)

- Do health professionals (doctor/ nurse/pharmacists) explain about the illness (what is the disease), treatment plan (medicine’s type, duration, the side effects of unnecessary antibiotics, etc.)? YES/NO/Sometimes (Explain) ____________________

Would this be helpful? How________________________________________________

- What difference does it make if you follow the doctors’ instructions in acute febrile illness? Why is this?

______________________________________________________________________

- Are antibiotics needed for every fever? YES/NO

Explain why______________________________________________

- Do you think antibiotics cure fever earlier than other medicine? YES/NO

Why?_________________________________________________________

- What do you think are the effects of taking antibiotics when you do not need them?

____________________________________________________

**Psychological**

*(Memory, attention and decision processes)*

- When you have bought medicine for your child, for a fever, how do you decide when and how the child should take the medicine?

_________________________________________________________________-

Opportunity

**Physical**

*(Environmental context and resources)*

- When your children are sick, are you able to bring them to Patan Hospital easily?

Where do you usually prefer to go when your child has an acute febrile illness? (Patan Hospital/Other Hospitals/Pharmacy/ home care)

- Explain why______________________________________________________________

If not in hospitals explain why do you visit there ____________________________________________(more time consuming/expensive/not satisfied with doctors)

- Do you go and buy medicines on our own when your child has acute febrile illness? YES/No/Sometimes

Why_________________________________________________________

**Social**

*(Social role/ influences)*

How does your friends and family behave when they are prescribed medicine from the clinic for fever? For example, do they follow the prescribers’ instructions? Do they complete the antibiotics as prescribed or do they buy antibiotics if not prescribed by doctors? Please elaborate by giving some examples.

----------------------------------------

- How do the expectations or actions of your family and friends influence which medicine you buy or if you follow the prescriber’s instructions?

______________________________________________________________________

- What is your opinion of those who take antibiotics even if not prescribed by doctors?

________________________________________________________

Motivation

**Reflective**

*(Beliefs about consequences)*

- What do you believe are the impacts to your child’s illness if you adhere to the prescribing regimen from the hospital?
- Would there be the any difference in outcome of your children illness when he/she is given antibiotic vs. when he/she is not given an antibiotic?

Explain why? ___________________________________________________________________

(Social role and identity)

- What is your opinion of someone who does not buy the medicine prescribed from the hospital or does not follow the instructions?

**Automatic**

*(Emotional response)*

What emotion do you feel if the doctor does not prescribe you an antibiotic, when your child has a fever?

Why____________________________________________

What emotion do you feel if the doctor prescribes you an antibiotic, when your child has a fever?

Why____________________________________________

*(Reinforcement)*

Do doctors remind encourage you in follow-up visits when you have adhered to what was prescribed? YES/NO

*(Social/professional roles/ identity)*

What are the factors that play an important role in adhering to the prescribed regimen?

______________________________________________________________________

What is the role of doctors_________________________?

What is the role of caregivers___________________________?

*(Routines and habits)*

What role do routines and habits play in which medicine you buy and where you buy it, or how the medicine is taken by the children?

How do they make it easier or more difficult?

____________________________________________________________________________
