# Supplementary material for: The facilitators of and barriers to antimicrobial use and misuse in Lalitpur, Nepal: a qualitative study
Source: BMC Public Health. 2024 May 2;24:1219. doi: 10.1186/s12889-024-18690-9 (PMC11067172; doi:10.1186/s12889-024-18690-9)
Supplement: Supplementary file 2 — Supplementary Material 2 [file 12889_2024_18690_MOESM2_ESM.docx]

**Supplementary File 2. Patients’ questionnaires for in depth interview**

**Demographic data**

1. Interviewers Name,
2. Questionnaire number
3. Date
4. Location of interview participants
5. Health Care Facility Name that the participant normally attends

**Screening question/Doer-non doer**

1. Role: Have you attended or intend to attend Patan Hospital when you have fever?

(Screen out respondents who answer ‘no’)

1. In the last 1 year did you have fever and go to Patan Hospital?

**General questions**

1. When you visit the Patan Hospital with a fever, what makes it easier/would make it easier for you to
2. Buy the medicine that the doctor in Patan Hospital prescribes
3. Take the medicine following the instructions from Patan Hospital
4. What makes it difficult to
   1. Buy the medicine that the doctor in Patan Hospital prescribes
   2. Take the medicine following the instructions from Patan Hospital

Normal behavior

The last time that you had fever and when you went to Patan Hospital

- Were you prescribed medicine?
- Did you buy medicines as instructed by physician? YES/NO
  - Why__________________________________________________________

What medicines were you given/did you buy?

___________________________________________________________

- Did you complete antibiotics as prescribed by the doctors YES/NO/NA
  - Why________________________________________
- Did you buy antibiotics if not prescribed by the doctors? YES/NO/NA
  - Why_________________________________________
- Did you come for a follow-up as instructed? YES/NO/NA
  - Why__________________________________________________________

*NA- not applicable

Capability

Knowledge

- Do you know what antibiotics are, and what they are used to treat? YES/NO

(If not to be explained by the interviewer)

- Do health professionals (doctor/ nurse/pharmacists) explain about the illness (what is the disease), treatment plan (medicine’s type, duration, the side effects of unnecessary antibiotics, etc.)? YES/NO

Would this be helpful? How________________________________________________

- What difference does it make if you follow the doctors’ instructions in acute febrile illness? Why is this?

______________________________________________________________________

- Are antibiotics needed for every fever? YES/NO

Explain why______________________________________________

- Do you think antibiotics cure fever earlier than other medicine? YES/NO

Why?_________________________________________________________

- What do you think are the effects of taking antibiotics when you do not need them?

____________________________________________________

**Psychological**

*(Memory, attention and decision processes)*

- When you have bought medicine for yourself, for a fever, how do you decide when and how you should take the medicines?

_________________________________________________________________-

Opportunity

**Physical**

*(Environmental context and resources)*

- When you are sick, are you able to come to Patan Hospital easily?
- Where do you usually prefer to go when you have an acute febrile illness? (Patan Hospital/Other Hospitals/Pharmacy/ home care)

Explain why______________________________________________________________

If not in hospitals explain why do you visit there ____________________________________________(more time consuming/expensive/not satisfied with doctors)

- Do you go and buy medicines on our own when you have an acute febrile illness? YES/No

Why_________________________________________________________

**Social**

*(Social role/ influences)*

- How do your friends and family behave when they are prescribed medicine from the clinic for fever? For example, do they follow the prescribers’ instructions? Do they complete the antibiotics as prescribed or do they buy antibiotics if not prescribed by doctors? Please elaborate by giving some examples.
- How do the expectations or actions of your family and friends influence which medicine you buy or if you follow the prescriber’s instructions?

______________________________________________________________________

- What is your opinion of those who take antibiotics even if not prescribed by doctors?

________________________________________________________

Motivation

**Reflective**

*(Beliefs about consequences)*

- What do you believe are the impacts if you adhere to the prescribing regimen from the hospital?
- Would there be the any difference in outcome of your illness when given antibiotic vs. when not given an antibiotic?

Explain why? ___________________________________________________________________

(Social role and identity)

- What is your opinion of someone who does not buy the medicine prescribed from the hospital or does not follow the instructions?

**Automatic**

*(Emotional response)*

What emotion do you feel if the doctor does not prescribe you an antibiotic, when you have a fever?

Why____________________________________________

What emotion do you feel if the doctor prescribes you an antibiotic, when you have a fever?

Why____________________________________________

*(Reinforcement)*

Do doctors remind encourage you in follow-up visits when you have adhered to what was prescribed? YES/NO

*(Social/professional roles/ identity)*

What are the factors that play an important role in adhering to the prescribed regimen?

______________________________________________________________________

What is the role of doctors_________________________?

What is the role of caregivers___________________________?

*(Routines and habits)*

What role do routines and habits play in which medicine you buy and where you buy it, or how the medicine is taken by you?

How do they make it easier or more difficult?

__________________________________________________________________________________
