# Supplementary material for: The facilitators of and barriers to antimicrobial use and misuse in Lalitpur, Nepal: a qualitative study
Source: BMC Public Health. 2024 May 2;24:1219. doi: 10.1186/s12889-024-18690-9 (PMC11067172; doi:10.1186/s12889-024-18690-9)
Supplement: Supplementary file 3 — Supplementary Material 3 [file 12889_2024_18690_MOESM3_ESM.docx]

**Supplementary File 3. Health care workers: FOCUS GROUP: DISCUSSION GUIDE**

Facilitator’s welcome, introduction and instructions to participants:

Welcome and thank you for volunteering to take part in this focus group discussion. We are very interested to hear your valuable opinion. You have been asked to participate as your point of view is important. I realize you are busy and I appreciate your time.

Introduction: This focus group discussion is designed to assess your current thoughts and feelings about (the communication of adherence to prescription in patients/guardians) or (Adherence to the use of algorithm and point of care tests). The information you give us is completely confidential, and we will not associate your name with anything you say in the focus group. We would like to audio tape the focus group discussion so that we can make sure to capture the thoughts, opinions, and ideas we hear from the group. No names will be attached to the focus groups. May I audio tape the discussion to facilitate its recollection? (If yes, switch on the recorder)

Anonymity: Despite being taped, I would like to assure you that the discussion will be anonymous. The tapes will be kept safely in a locked facility until they are transcribed word for word, then they will be destroyed. The transcribed notes of the focus group will contain no information that would allow individual subjects to be linked to specific statements. You should try to answer and comment as accurately and truthfully as possible. I and the other focus group participants would appreciate it if you would refrain from discussing the comments of other group members outside the focus group. If there are any questions or discussions that you do not wish to answer or participate in, you do not have to do so; however please try to answer and be as involved as possible. You may refuse to answer any question or withdraw from the study at anytime

- Logistics:
- Focus group will last about one hour
- Feel free to move around
- Where is the bathroom? Exit?
- Help yourself to refreshments.
- Ground rules:
- The most important rule is that only one person speaks at a time. There may be a temptation to jump in when someone is talking but please wait until they have finished.
- There are no right or wrong answers
- You do not have to speak in any particular order
- When you do have something to say, please do so. There are many of you in the group and it is important that I obtain the views of each of you
- You do not have to agree with the views of other people in the group
- Does anyone have any questions?
- OK, let’s begin
- Introduction

Introduce the facilitator and the note taker, and send the Sign-In Sheet with a few quick demographic questions (age, gender, professional title/background, and years of experience with professional title) around to the group while facilitator is introducing the focus group.

**Questions: Adherence to prescription**

- Introductory question

I am just going to give you a couple of minutes to think about your experience of communicating with the patients?

- Guiding questions
- What makes it easier/would make it easier for you to

Communicate to patients/caregivers about adherence to the prescription

- What makes it difficult to
  - communicate to patients/caregivers about adherence to the prescription
- **Probes for discussion: for communicating with patients to adhere to treatment regimen.**
- Knowledge and skills
  - How do you decide what and how to communicate?
- Physical opportunity:

How and where do explain your treatment plan to patients with different backgrounds? (What is the illness, how long to take medicine, when to come to follow up?

How does your available time, or location, influence how you communicate treatment plans to patients?

- Expectations of others:

What are the expectations of others (in the community who are not medical professionals) that influence what or how you communicate?

- Expectations of other medical professionals
  - What are the expectations of other medical professionals that influence what or how you communicate? Does this make it easier or more difficult?
- Belief in Consequences:
  - What do you believe are the consequences of communicating to patients/caregivers about adherence to the prescription?
- Routines and habits:
  - What role do routines and habits play in your communication to patients/caregivers about adherence to the prescription? Do they/would they make it easier or more difficult?
- Concluding question
- Of all the things we’ve discussed today, what would you say are the most important issues you would like to express about the communication to patients/caregivers about adherence to the prescription?
