# Supplementary material for: The facilitators of and barriers to antimicrobial use and misuse in Lalitpur, Nepal: a qualitative study
Source: BMC Public Health. 2024 May 2;24:1219. doi: 10.1186/s12889-024-18690-9 (PMC11067172; doi:10.1186/s12889-024-18690-9)
Supplement: Supplementary file 4 — Supplementary Material 4 [file 12889_2024_18690_MOESM4_ESM.docx]

**Supplementary File 4. Physician questionnaires on adherence to prescribed treatment**

**Demographic data**

1. Interviewers Name,
2. Questionnaire number
3. Date
4. Health Care Facility Name
5. What is your professional title/background (e.g. Doctor, Nurse, etc)
6. How many years’ experience with this professional title/background have you had?

**Screening question/Doer-non doer**

1. Role at the clinic: Do you have responsibility to
   1. communicate to patients/caregivers about adherence to the prescription

(Screen out respondents that are not responsible)

1. In the last 2 weeks did you
   1. communicate to patients/caregivers about adherence to the prescription

**General questions**

1. What makes it easier/would make it easier for you to
   1. communicate to patients/caregivers about adherence to the prescription
2. What makes it difficult to
   1. communicate to patients/caregivers about adherence to the prescription

Capability

**Psychological**

*(Memory, attention and decision processes)*

How do you decide what to communicate to patients/caregivers about adherence to prescription in acute febrile illness?

____________________________________________________________________________

Knowledge

Why it is important is to communicate to patients/caregivers about adherence to prescription in acute febrile illness?

___________________________________________________________________________

Knowledge/Skills *(instruct not to take antibiotic in every fever)*

- Do you instruct to buy medicines as prescribed? Yes/No
- Why________________________________________________________________

*(Further evaluate with two indirect questions)*

- Do you instruct to specifically to complete antibiotics as prescribed in acute febrile illness? YES/NO

Explain why________________________________________

- Do you instruct to specifically not buy antibiotics if not prescribed in acute febrile illness? YES/NO

Explain why_________________________________________

Knowledge/skills – *(Explain the disease process and management)*

Do you explain about course of disease, management plan and prescription while managing patients/caregivers with acute febrile illness? YES/NO

Explain why______________________________________________

What are the most important communication skills that a doctor should have to make patients adhere to prescription of acute febrile illness?

________________________________________________________________________

Knowledge/Skills (to ask to come for follow-up as instructed)

- Do you advise to come for a follow-up as instructed? YES/NO
- Why___________________________________________________________________

What factors of the patients/caregivers do you think will be important in making patients understand about the importance of adhering to the prescribed regimen?

____________________________________________________________________________

Opportunity

**Physical**

*(Environmental context and resources)*

How do explain your treatment plan to patients/caregivers with different socioeconomic backgrounds? (What is the illness, how long to take medicine, when to come to follow-up?)

How do you manage your time to explain this during the clinical encounter? ___________________________________________________________

**Social**

*(Social role/ influences)*

Do your colleagues advise patients to take medicine as prescribed in acute febrile illness?

Do your seniors advise patients with acute febrile illness to take medicine as prescribed?

Do your colleagues/seniors advise patients with acute febrile illness to not to take antibiotics if not prescribed?

Do other outpatient staffs (nurse/Community health worker) advise patients with acute febrile illness to take medicine as prescribed?

Do patients/caregivers except the rationale to why they should adhere to prescribed regimen?

What is your opinion of those doctors who do not ask patients to adhere to the prescription?

How do the expectations patients / other doctors affect your decision to ask for adherence to prescribed medicines?

Motivation

**Reflective**

*(Beliefs about capabilities and optimism)*

Do you think that patients will adhere to the prescribing regimen if you explain them more clearly? YES/NO

Why?

*(Beliefs about consequences)*

What do you believe are the consequences of adherence from giving the instructions vs. not giving the instructions by a health care worker?

_________________________________________________________________________

*(Intentions)*

Do you intend to ask patients/caregivers to adhere to prescribed regimen for acute febrile illness?

**Automatic**

*(Emotional response)*

How do you feel when patients/caregivers adhere to the prescribed regimen?

Why____________________________________________

How do you feel when patients/caregivers do not adhere to the prescribed regimen?

Why____________________________________________

*(Reinforcement)*

Do you encourage in follow-up visits when they have adhered to what was prescribed? YES/NO

Why?__________________________________

*(Social/professional roles/ identity)*

What are the factors that play important role in adhering to the prescribed regimen?

______________________________________________________________________

What is the role of doctors_________________________?

What is the role of caregivers/patients___________________________?

*(Routines and habits)*

What role do routines and habits play in your communication to patients/caregivers about adherence to the prescription?

____________________________________________________________________________

How do they make it easier or more difficult?
