# Supplementary material for: The facilitators of and barriers to antimicrobial use and misuse in Lalitpur, Nepal: a qualitative study
Source: BMC Public Health. 2024 May 2;24:1219. doi: 10.1186/s12889-024-18690-9 (PMC11067172; doi:10.1186/s12889-024-18690-9)
Supplement: Supplementary file 5 — Supplementary Material 5 [file 12889_2024_18690_MOESM5_ESM.docx]

# **Supplementary File 5. Day 0 Patient/Caregiver questionnaire**

**The topics are:**

- **Where does the patient/caregiver normally buy their medicine? Do they normally buy the medicine that is prescribed or buy something else?**
- **Does the patient/caregiver they normally take the medicine as prescribed e.g. the frequency and duration stated in the prescription?**

**Questions**

- Could you please describe where you like to buy (or receive) medicine from?
- Do you normally buy (or receive) the medicine on the prescription, or something else? Could you share your motivations (what makes you) for buying something else?
- And could you tell me a bit more about this medicine you buy, for how long do you usually take it (linked to next question), the type (e.g. tablets or liquid), perhaps you remember the name…? (this should tell us if they complete the treatment duration and link it to next question)
- Could you please describe when you usually finish taking the medicines? Who does usually decide when to finish? Why? What do you do with any leftovers (if any, depending on previous answers)?

# Day 7 Patient/Caregiver questionnaire

**The topics are:**

1. **Did the patient/caregiver adhere to the prescription and why,**
   1. **Did they buy the medicine that was prescribed, (or receive the medicine that was prescribed for health facilities where the patient does not pay for medicine)**
   2. **Did they complete the course as per the prescription?**
2. **Do they intend to ask for antibiotics the next time they/their child comes to the clinic with a fever?**
3. **Did the patient/caregiver knowledge and awareness change due to the communication received on Day 0**

**Questions**

- Can you please describe what you did after you were given the prescription a week ago? (Prompt to find out what was bought, from where?) Why did you go to that place to get the medicine? Can you share with me why you bought this particular medicine?
- Can you please describe to me what you did once you had the medicine? How did you take the medicine/give the medicine to your child? Did you do anything to help them take the medicine? (When applicable)...Explain to me how many pills you took/your child took each day.
- Could you please describe when you finish taking the medicines? Who decided when to finish? Why? What did you do with any leftovers (if any, depending on previous answers)
- Examine the packaging (Patients/caregivers are requested to bring in the medicine packaging) to identify how many pills have been taken and cross-check with their answer, probe to clarify if needed.
- Can you please explain to me what you expect to get if you come to the clinic in the future with a fever/bring your child to the clinic with a fever?
- Can you please share with me what you thought about the message you were given about the medicine that you were told when you saw the healthcare worker a week ago?
