# Supplementary figures and images for: The facilitators of and barriers to antimicrobial use and misuse in Lalitpur, Nepal: a qualitative study
Source: BMC Public Health. 2024 May 2;24:1219. doi: 10.1186/s12889-024-18690-9 (PMC11067172; doi:10.1186/s12889-024-18690-9)

**Supplementary File 6. ‘Ask Me’ brochure**


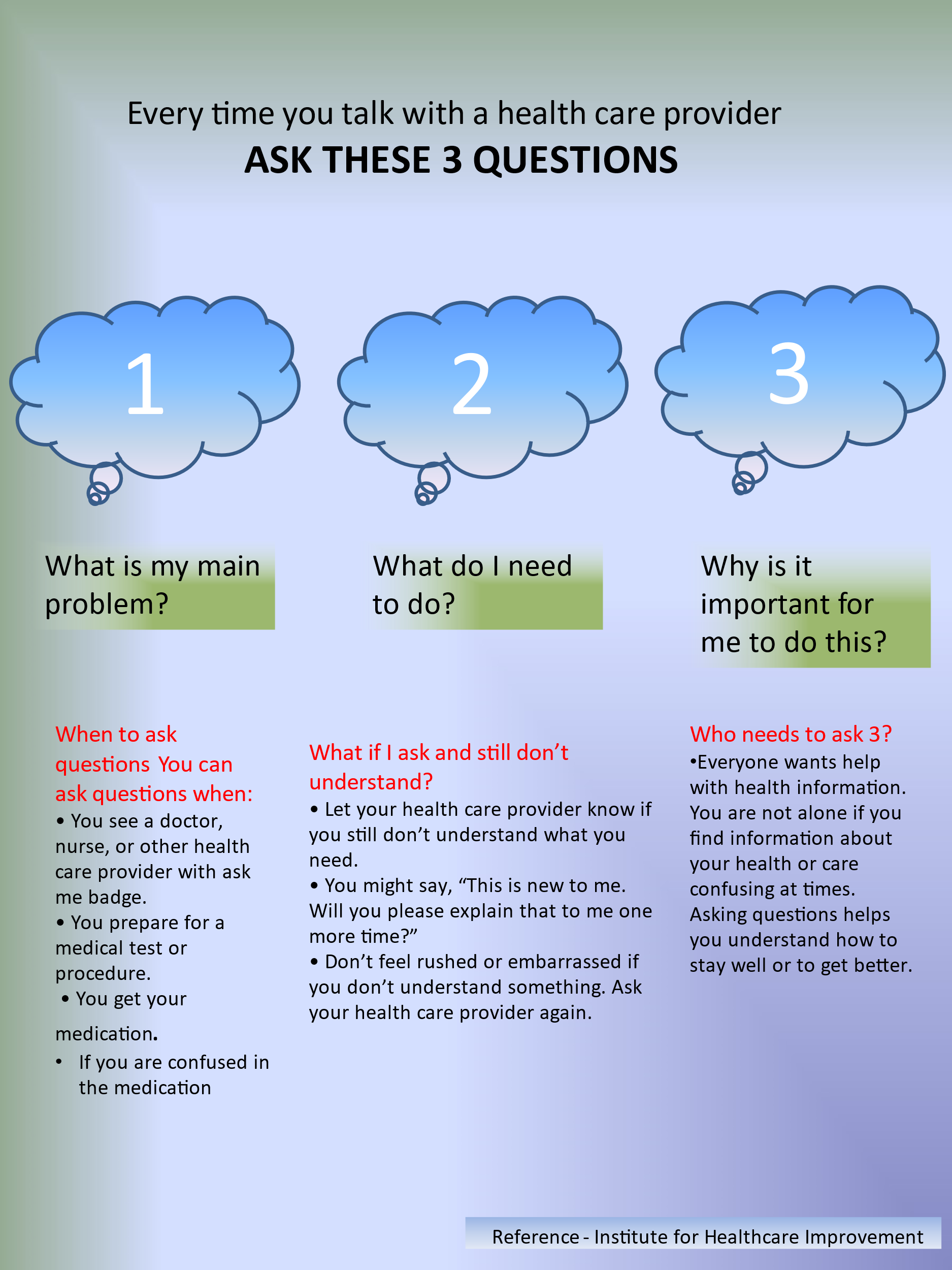


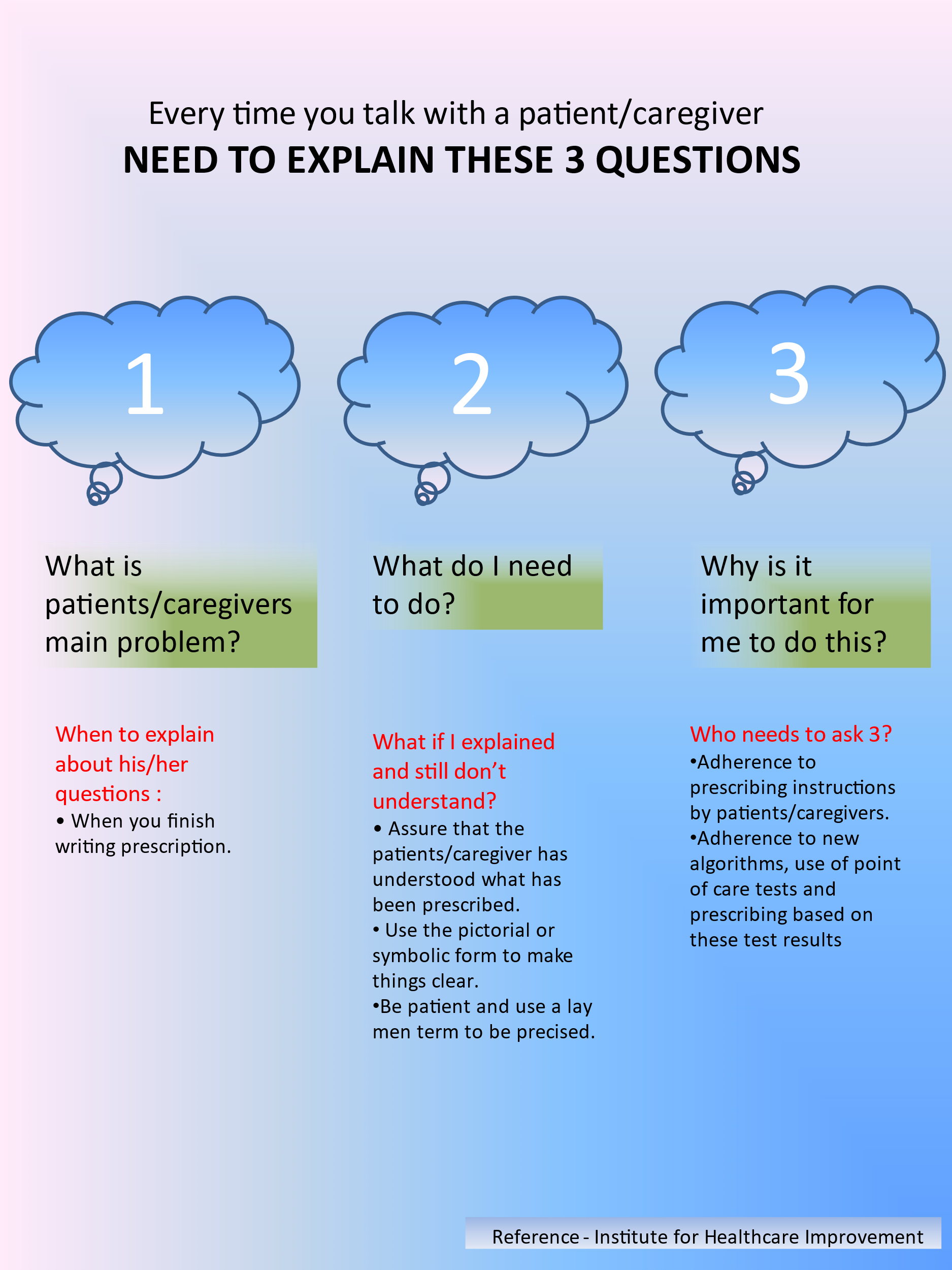

Supplement: Supplementary file 6 — Supplementary Material 6 [file 12889_2024_18690_MOESM6_ESM.docx]

**Supplementary File 7. ‘Ask Me’ table calendar for patients and caregivers**


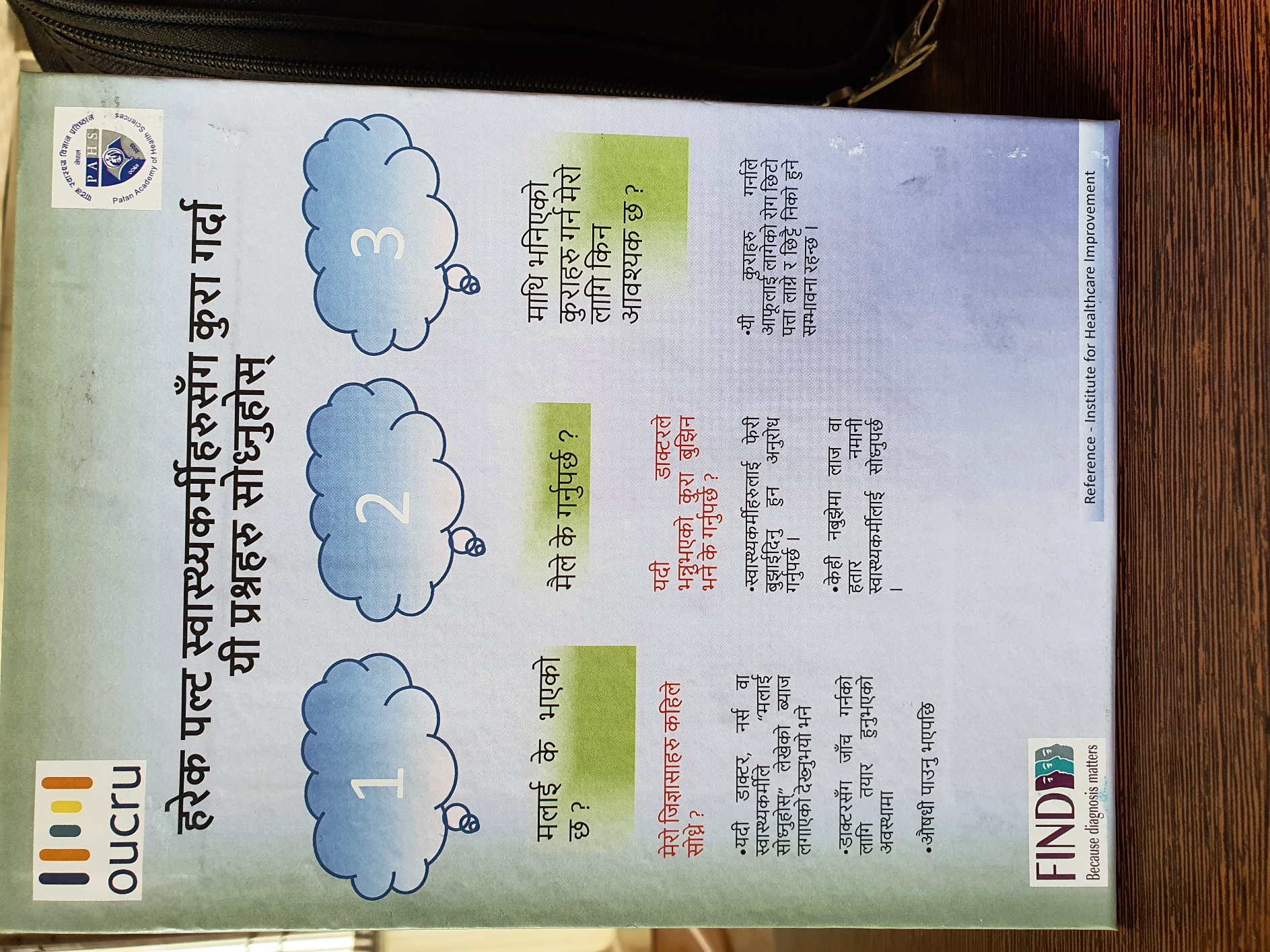

Supplement: Supplementary file 7 — Supplementary Material 7 [file 12889_2024_18690_MOESM7_ESM.docx]

**Supplementary File 8. Digital display boards**


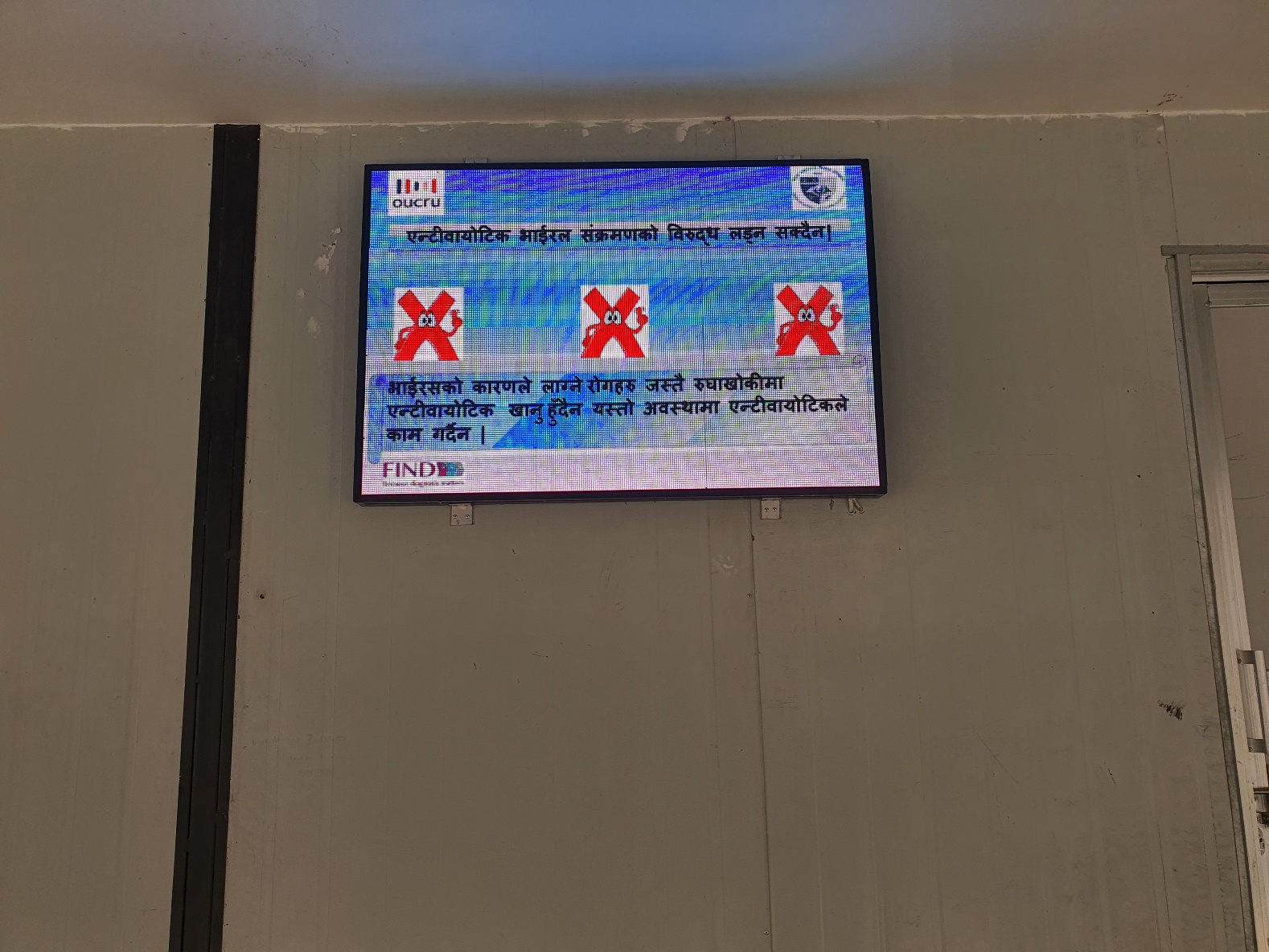

Supplement: Supplementary file 8 — Supplementary Material 8 [file 12889_2024_18690_MOESM8_ESM.docx]

**Supplementary File 9. Digital display boards [2]**


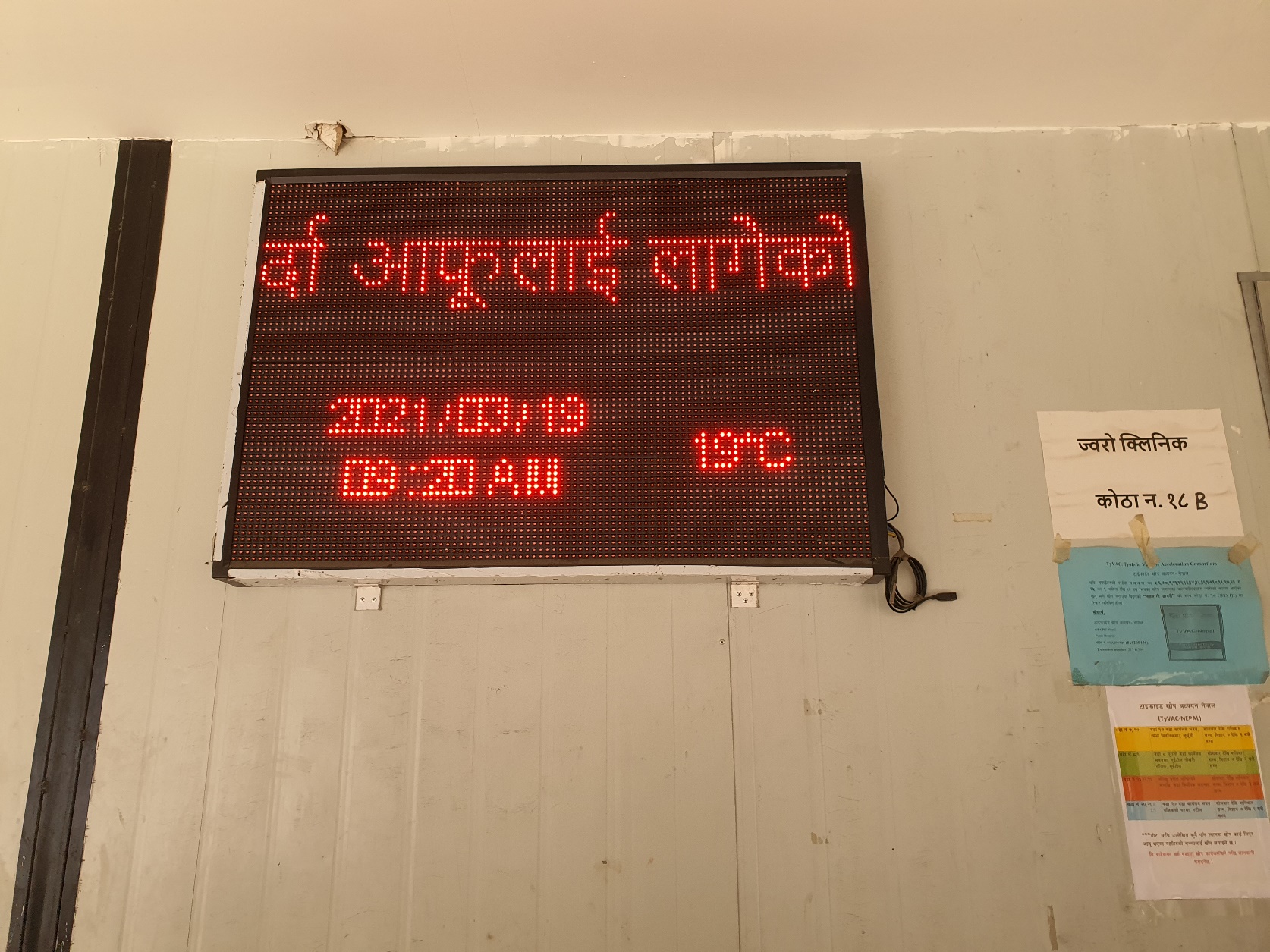

Supplement: Supplementary file 9 — Supplementary Material 9 [file 12889_2024_18690_MOESM9_ESM.docx]

**Supplementary File 10. Medical bag**


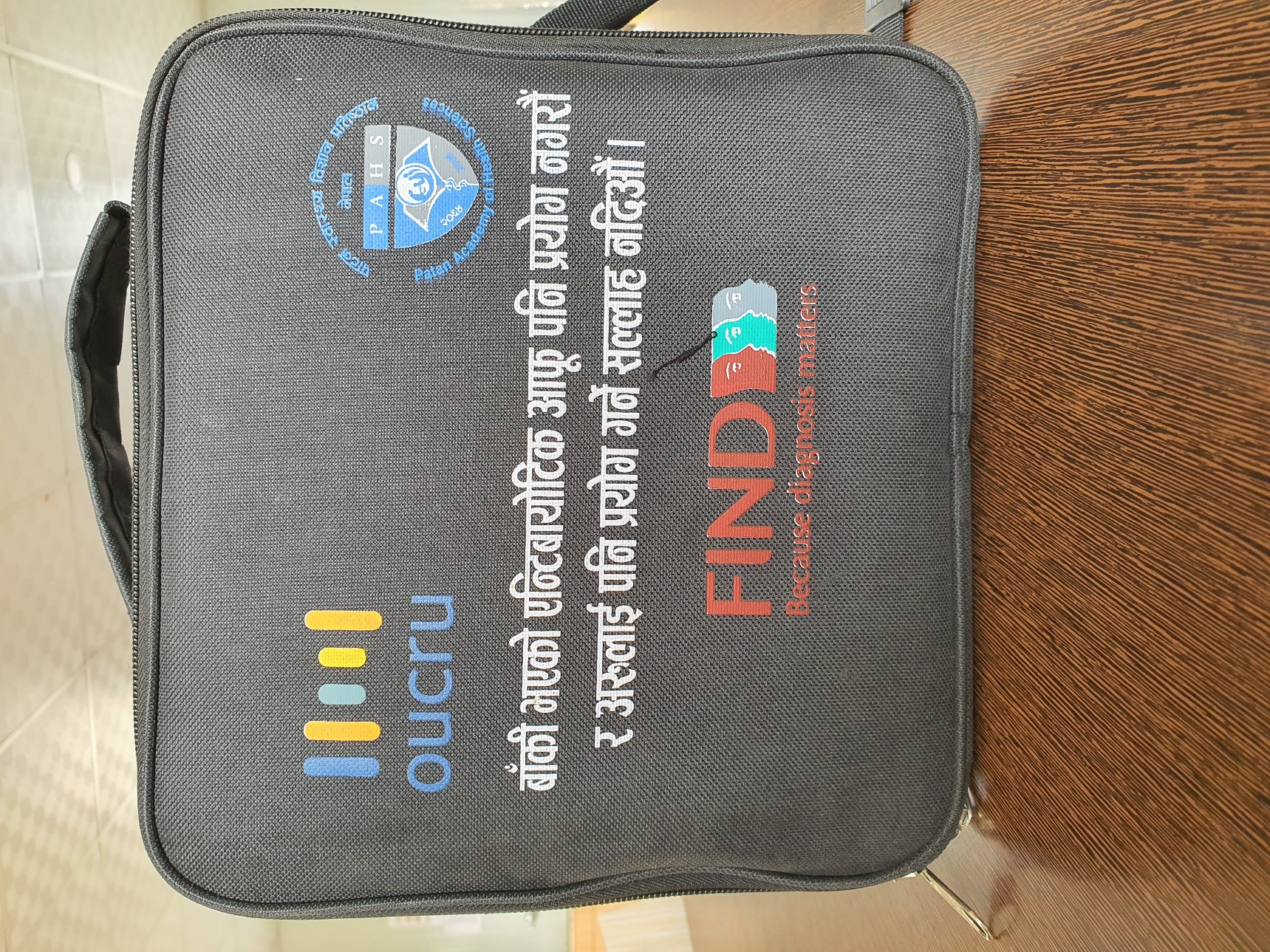

Supplement: Supplementary file 10 — Supplementary Material 10 [file 12889_2024_18690_MOESM10_ESM.docx]

**Supplementary File 11. Medication reminder chart for patients and caregivers**


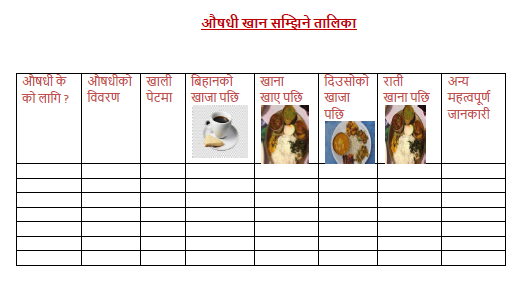

Supplement: Supplementary file 11 — Supplementary Material 11 [file 12889_2024_18690_MOESM11_ESM.docx]

**Supplementary File 12. Misconceptions about antibiotics**


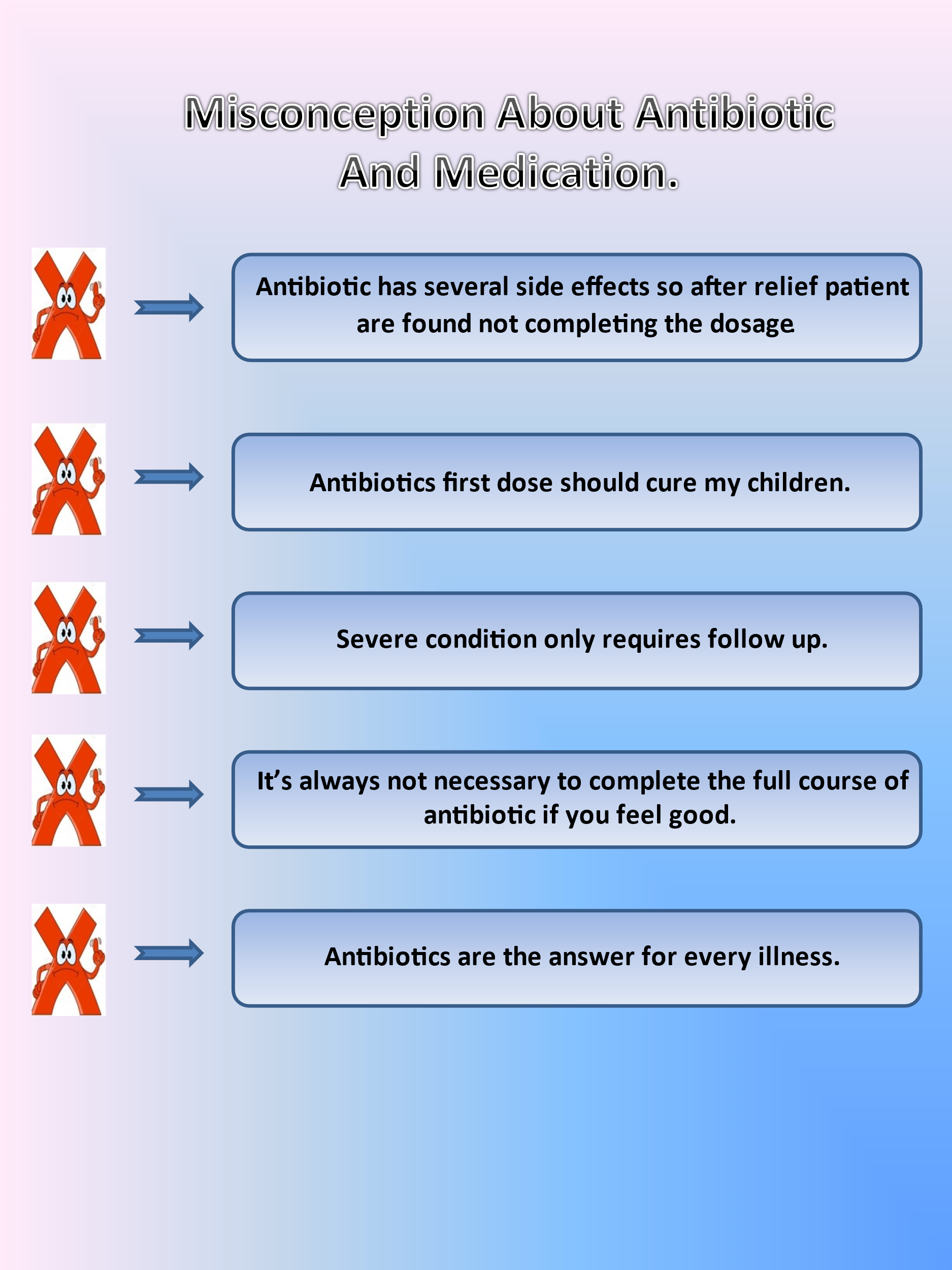

Supplement: Supplementary file 12 — Supplementary Material 12 [file 12889_2024_18690_MOESM12_ESM.docx]

**Supplementary File 13. Take home messages for patients**


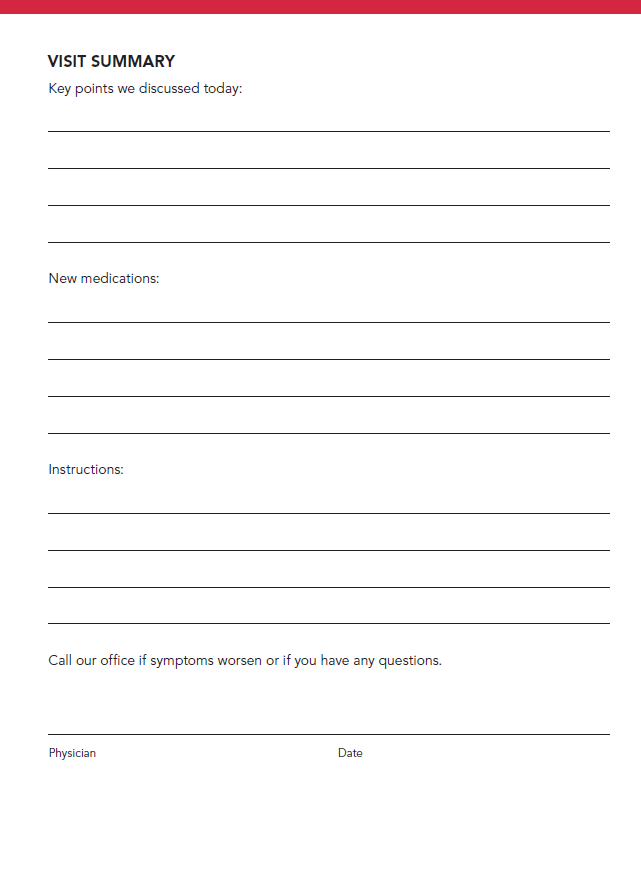

Supplement: Supplementary file 13 — Supplementary Material 13 [file 12889_2024_18690_MOESM13_ESM.docx]
