# Supplementary material for: The facilitators of and barriers to antimicrobial use and misuse in Lalitpur, Nepal: a qualitative study
Source: BMC Public Health. 2024 May 2;24:1219. doi: 10.1186/s12889-024-18690-9 (PMC11067172; doi:10.1186/s12889-024-18690-9)
Supplement: Supplementary file 14 — Supplementary Material 14 [file 12889_2024_18690_MOESM14_ESM.docx]

**Supplementary File 14. Verbal messages for health care providers**

Verbal Message for Health Care Providers

# How to communicate well

1. Make the patient comfortable and assure them that they are here to help them
   1. If a patient is particularly panicked call nurse for support (e.g. distracting the child)
2. Be patient and ensure you listen to the patient.
3. Sometime doctors can get irritated or angry, how should we deal with this if it happens? (for discussion in training)
4. Use of Simple Language: Avoid medical terms, use of laymen terms
5. Be consistent – ensure all messages are provided for all patients
6. Ensure that writing of the prescription is legible
7. Communicating with the older/elderly patients or Caregivers.We can use the following strategies,
   1. **Ask Instead of making Assumption**-
      1. Similarly, ask questions instead of making assumptions when it comes to your actions in relation to the older adult.
      2. Otherwise, explain why it’s important for you to do what you need to do (in most cases for the sake of senior’s health and well-being).
      3. Instead, use statements that begin with “I,” “It,” “We,” “Let’s,” and “This,” to convey messages. For example, “It’s important to take your medicine.” This doctor’s appointment is very important.
      4. (Note- “These types of statements compel the older adult to be more open to what you have to say, encourage listening, and reduce the possibility of fight, flight, or freeze responses.)
      5. Give them a little extra time in counseling and while prescribing use the pictures /figures so that they can know which medicine to take.
8. If they do not understand ask them to note it down in their way so that they do not forget or use symbol.
9. Followup with the patient to get reassurance that they do understand the prescription
10. If patients show some difficultly in understanding the prescription, use the signs/pictures
11. By the end of the patient visit ensure the 3 questions on the calendar have been asked, answered and understood.
12. Call nurse to help in translation to and from Newari
13. Shared phone numbers so patients can ask questions later

Highlighted are physical items

# Messages Day 0 (a) – first interaction

Doctor to introduce themselves, to make the patient comfortable and help build a trusting relationship with the patient, include details of:

1. Who you are
2. Experience (e.g. I’ve been a doctor for x years..’, or ‘I trained at …’)
3. Small talk/icebreaking

# Messages Day 0 (b)- return after test results

1. Test results and health condition/diagnosis
2. Prescription
   1. What medicine is prescribed
   2. Ensure that the name of the drug is clearly written (not illegible),
   3. Dosage and timing
      1. Explain clear information about the dosages – at what time to take the medicine, and how long between doses (check what this means for the time of each dose), and for how many days;
      2. Explain clearly about taking before/with/after meals and if there are foods that should be avoided when taking the medicine. (Complete the pictures if helpful)
      3. Get reassure from the patient that they understand exactly what that means
   4. Why (this medicine)
      1. If you take this medicine as I have recommended you should recover.
      2. Difference between Antibiotic and Non –Antibiotic: Antibiotics vs. non antibiotic
         1. Stress the health benefits of taking antibiotic when needed with examples.
         2. The side effects of antibiotics when not needed.
         3. Make them clear when antibiotic is needed and when not. Clearly, explain how the only antibiotics work against bacterial infections and other medicine does not make you better, explain (simply) about drug resistance.
      3. Clearly make them understand about the health condition and why is it important (and how important) for them do this? Reassure/ Use of Pictures.
         1. (Case 1)Incase they do not need antibiotics, explain them why an antibiotic is needed or not; the side effects of taking antibiotics when not needed; highlight cost differences and time consequences (e.g. if need to travel to a different pharmacy)
         2. (Case 2.) Advice to patient if they might forget
            1. Set an alarm/ask your family member (particularly for elderly patients)
            2. Make a note for them or ask them to note down in there way
   5. Why (dose and complete course)
      - 1. Explain to the patient the likely consequences (e.g. effects on recovery) if they do not take the medicine at the time advised and complete.
           1. Do not stop taking the medicine before the end of the course, even if you feel better or if someone says “its fate” “you don’t need to take the medicine”.
           2. I know everyone has a busy life now, but its important that you take the medicine on time. If you forget a dose and add *instructions what to do if miss a dose*…(discuss in training)
           3. When the patient is a child – ask the parent/caregiver: Does the child have difficultly taking medicine sometimes? If yes, provide information on how to make it easier. (discuss in training)
   6. Provide an example of what the prescriber does for the patients to adhere the prescription, e.g. ‘When I am sick with the flu like you are, I take one pill in the morning and one in the evening for 5 days and I really take care that I do not miss even one of these pills or times.’ (note this should include the medicine, dosage/timing and completion)
   7. Side effects: Clear information about the side effects
      1. **Explain if/when to continue with medicine or when to stop and return to the hospital.**
      2. *Repeat message about health benefits of completing the course*
      3. Ask the patients or caregivers to repeat the instructions back to the prescriber and if they have any side effects visit the doctor
   8. Ask the patient about the 3 questions – have they been asked, answered and understood? If not repeat/respond
   9. ask the patient/caregiver to show the prescription to the dispenser and check that they have given you the one named on the prescription
