# Supplementary material for: The facilitators of and barriers to antimicrobial use and misuse in Lalitpur, Nepal: a qualitative study
Source: BMC Public Health. 2024 May 2;24:1219. doi: 10.1186/s12889-024-18690-9 (PMC11067172; doi:10.1186/s12889-024-18690-9)
Supplement: Supplementary file 15 — Supplementary Material 15 [file 12889_2024_18690_MOESM15_ESM.docx]

**Supplementary File 15: Verbal messages for nurses and other Research Teams**

Verbal Message for Nurses

(Make the patient comfortable and assure them that they are here to help them)

1. **Providing / Explaining the Patients/ Caregivers the Communication Package**
2. Reassure regarding the doctors advice and make sure they understood
3. Stress the health benefits of taking antibiotic when needed with examples.
4. Making them understand what will happen if they take the medicine without the doctor advice.
5. Reassure regarding the side effects of antibiotics.
6. Explain the effects on recovery for the patient/child if you take the medicine at the specified time of the day and do not miss.
7. Make them clear regarding the completion of medicine dose
8. Importance of follow up.
9. If they have any problems and queries provide them the contact numbers.
